# Increases in Prevalent Depressed Mood and Suicidal Ideation among Workers during the COVID-19 Pandemic—Findings from the California Health Interview Survey

**DOI:** 10.3390/ijerph20021253

**Published:** 2023-01-10

**Authors:** Kathryn Gibb, David Pham Bui, Ximena P. Vergara

**Affiliations:** 1 Occupational Health Branch, California Department of Public Health, Richmond, CA 94804, USA; 2 Public Health Institute, Oakland, CA 91746, USA; 3 Heluna Health, City of Industry, CA 91746, USA

**Keywords:** mental health, COVID-19, depression, suicidal ideation, occupational

## Abstract

Limited data exist on COVID-19’s mental health impact on non-healthcare workers. We estimated the prevalence of depressed mood and suicidal ideation experienced in the past year among California workers and assessed whether the prevalence changed during the COVID-19 pandemic. We analyzed 2013–2020 California Health Interview Survey data using survey-weighted methods to assess the change in the prevalence of depressed mood and suicidal ideation from 2019 to 2020 for working adults by demographics and occupational groups. We used trend-adjusted quasi-Poisson regressions and report rate ratios (RR), comparing the prevalence of outcomes during 2020 to the pre-pandemic period (2013–2019). We identified priority occupation groups with a higher-than-average outcome prevalence in 2020 and rate increases after adjusting for pre-pandemic trends. Our analysis included 168,768 respondents, of which 65% were workers. Production and service workers were the priority occupation groups for depressed mood (RR: 1.46, CI: 1.1–1.9; RR: 1.23, CI: 1.1–1.4) and suicidal ideation (RR: 1.86, CI: 1.0–3.6; RR: 1.47, CI: 1.1–1.9). Workers aged 45–65 years experienced over a 30% relative increase in both outcomes from 2019 to 2020. Depressed mood and suicidal ideation in the past year increased for production, service, and older workers during the pandemic. These groups should be considered for mental health interventions.

## 1. Introduction

Depression and anxiety increased in the United States during the early months of the COVID-19 pandemic for the general public [1,2]. Necessary public health measures such as quarantine and social distancing contributed to loneliness and isolation, which, in turn, adversely impacted mental health [3]. Previous public health emergencies, including the 2003 severe acute respiratory syndrome (SARS) and the 1918 influenza pandemics, were associated with increases in suicide, highlighting the unintended consequences of a pandemic response [4]. From a public health perspective, addressing poor mental health is critical as it is associated with prevalent chronic diseases including cardiovascular disease, diabetes, and obesity [5]. Mental health is also an economic concern, as poor mental health is associated with disability, lost productivity, absenteeism, and unemployment [6,7,8].

Workers have faced additional burdens during the COVID-19 pandemic, including job and income loss [9], which are associated with poor self-rated health and depressive symptoms [10]. High rates of burnout, anxiety, and post-traumatic stress among healthcare workers have been described [11]. Multiple studies found that essential, in-person workers have faced adverse mental health impacts due to fear of contracting COVID-19 at work, fear of infecting loved ones at home, and lack of adequate personal protective equipment [1,12,13]. Remote workers faced disruptions to their work–life balance, particularly for working parents with limited access to childcare and for workers without dedicated home office spaces [14]. While many surveys have examined the prevalence of depression or anxiety throughout the pandemic, few studies [15] have accounted for existing trends in mental health outcomes.

The strain of the pandemic on mental health may be unevenly distributed across worker groups. For instance, adults with disabilities report more frequent mental distress than adults without disabilities [16]; it is possible that these findings extend to workers. Furthermore, increases in suicidal ideation during the COVID-19 pandemic, particularly among younger people were evident, which may indicate age-related disparities among workers [1]. Understanding which workers have had increases in adverse mental health outcomes during the pandemic is critical to inform policymaking for the current pandemic response and future pandemic mental health preparedness. To address this knowledge gap, we used California Health Interview Survey (CHIS) data to assess the changes in prevalent depressed mood and suicidal ideation among California workers associated with the COVID-19 pandemic. We built upon existing research by utilizing a repeated cross-sectional approach to account for pre-pandemic trends in mental health outcomes to better isolate the impact of the pandemic.

## 2. Materials and Methods

### 2.1. Study Design, Survey Instrument, and Study Population

We used a repeated cross-sectional study using data from the adult version of CHIS from 2013 to 2020 (CHIS began collecting occupation data in 2013). CHIS is the largest state health survey in the United States and is designed to be representative of California’s noninstitutionalized population. CHIS surveys over 20,000 households annually and had an adult response rate of 11.6% for 2019–2020 [17]. CHIS was administered by phone until 2019 when it transitioned to allowing participants to complete the survey by web or telephone. In addition to demographic information and mental health outcomes, CHIS questionnaires collect data on topics including chronic diseases, health behavior, healthcare service utilization, health insurance coverage, and housing. Further details on CHIS questionnaires, survey methodology, and sample design can be found on the University of California, Los Angeles Center for Health Policy website [17].

Our study population consisted of California workers, defined as CHIS respondents (>17 years old) who reported they were currently or usually employed at the time of survey, regardless of full- or part-time status. Because CHIS was not designed to sample persons residing in institutional settings including military bases, we excluded military workers from the analyses.

### 2.2. Outcome Variables

We examined two adverse mental health outcomes: depressed mood in the past 30 days and reported suicidal ideation in the past year.

For evaluating prevalent depressed mood, we used the CHIS question, “About how often during the past 30 days did you feel so depressed that nothing could cheer you up?” For our primary analysis, we analyzed depressed mood as a binary variable and considered respondents who said they felt depressed all, most, some, or a little of the time during the past 30 days as having prevalent depressed mood. As a sensitivity analysis, we assessed depressed mood among respondents who said they felt depressed (1) all or most of the time (more frequent) and (2) some or a little of the time (less frequent) to determine if changes in depressed mood were driven by respondents that felt depressed more or less frequently.

Suicidal ideation in the past year was asked of the 1661 workers who said they ever thought seriously about committing suicide. Prevalent suicidal ideation in the past year was analyzed as a binary variable and based on the response to “Have you seriously thought about committing suicide in the past 12 months?” Adults who responded affirmatively were considered as having prevalent suicidal ideation.

### 2.3. Demographics

We assessed mental health outcomes by age group (18–29, 30–44, 45–64, 65+ years), self-reported sex (male/female), race/ethnicity, annual household income, educational attainment, industry, occupation, and whether the respondent lost their job or had reduced hours due to COVID-19. We also assessed outcomes among workers with disabilities, specifically those self-identifying as “blind or deaf,” or reporting “a severe vision or hearing impairment.” Indicators for difficulty concentrating, dressing or bathing, and doing errands alone were available starting in 2019 and were included in a subset of unadjusted analyses.

CHIS’s self-reported race/ethnicity variable includes Hispanic, Non-Hispanic (NH) White, NH African American, NH American Indian/Alaskan native, NH Asian, NH Native Hawaii/Pacific Islander, and two or more race categories. For analyses, we collapsed the American Indian/Alaska native, Native Hawaiian/Pacific Islander, and two or more races to prevent unintended disclosure due to small cell sizes.

CHIS used the National Institute for Occupational Safety and Health’s (NIOSH) Industry and Occupation Computerized Coding System (NIOCCS) to code open-ended industry and occupation responses to standardized Census 2010 Occupation Classification Schema [18]. Codes were grouped into major occupation and industry group categories using the Census classification system. All results are presented at the major occupation and industry group level.

### 2.4. Pandemic Indicator and Time Variables

In 2020, CHIS collected data between 9 March and 31 October, resulting in 96% of the 2020 CHIS data being collected after the first stay-at-home orders in California [19]. We created a pandemic indicator variable, differentiating the 2020 pandemic year from other non-pandemic years. This indicator variable was used in analyses to estimate changes in the prevalence of mental health outcomes during the pandemic compared to prior years.

### 2.5. Statistical Analysis

We used CHIS-supplied replicate survey weights and jackknife method variance estimation to compute standard errors and prevalence estimates. For each mental health outcome, we generated survey-weighted annual prevalence estimates and Korn–Graubard 95% confidence intervals (CI); estimates were generated for each demographic of interest. All reported estimates are weighted unless indicated otherwise.

To estimate the impact of the COVID-19 pandemic on mental health, we used survey-weighted *t*-tests to assess the absolute change in prevalence of each mental health outcome from 2019 to 2020 for all adults by working status, demographics, industry, occupation, and whether they lost their job or had their hours reduced due to COVID-19.

Since changes in adverse mental health outcomes from 2019 to 2020 may partially be due to historic trends, we used trend-adjusted quasi-Poisson regression models to adjust for prior trends and better isolate the impact of the pandemic on mental health. Quasi-Poisson regression is a generalization of Poisson regression that relaxes the constraint that the outcome variance must equal the mean and is commonly used to model overdispersed count data [20]. We modeled the count of the mental health outcomes as the dependent variable and our pandemic indicator as the independent variable as both linear and curvilinear. Time was modeled either quadratically or linearly in our final models, which we determined using log-likelihood ratio tests. When time (i.e., trend) coefficients were not statistically significant (*p* > 0.05), we reported results from an intercept-only model. The exponentiated coefficient for the pandemic indicator variable in our models can be interpreted as an adjusted prevalence rate ratio (RR), estimating the relative change in prevalence of the mental health outcomes during the pandemic compared with the expected prevalence had pre-pandemic trends continued. We report adjusted RRs with 95% CIs for all modeled outcomes.

### 2.6. Identifying Priority Occupation Groups

To aid framing our results, we defined priority occupation groups as those with higher-than-average prevalence rates of adverse mental health outcomes in 2020 and higher-than-average rate increases after adjusting for pre-pandemic trends. Statistical analyses were conducted using R Statistical Software (v 4.1.0; R Core Team 2022, R Foundation for Statistical Computing, Vienna, Austria) and the R ‘survey’ package [21].

### 2.7. Ethical Considerations

The California Health and Human Services Agency’s Committee for the Protection of Human Subjects determined this project to be non-research because the activities involved public health practice/surveillance to inform the state’s COVID-19 emergency response. CHIS data files were accessed through the California Department of Public Health (CDPH) Center for Health Statistics and Informatics, which maintains the confidential funder files provided to CDPH.

## 3. Results

### 3.1. Study Population

From 2013 to 2020, CHIS surveyed 168,768 adults, of which 87,703 were workers. In 2020, 21,949 adults were surveyed, of which 12,982 (59%, unweighted) were workers. Table 1 shows the sociodemographic characteristics of our study population in 2020; previous years can be found in the Supplemental Materials Table S5. Compared to nonworkers, workers were middle-aged (predominantly between 30–64 years), male, Hispanic/Latino, and from high-earning households (annual household income > $100,000).

### 3.2. Depressed Mood

**Figure 1:**
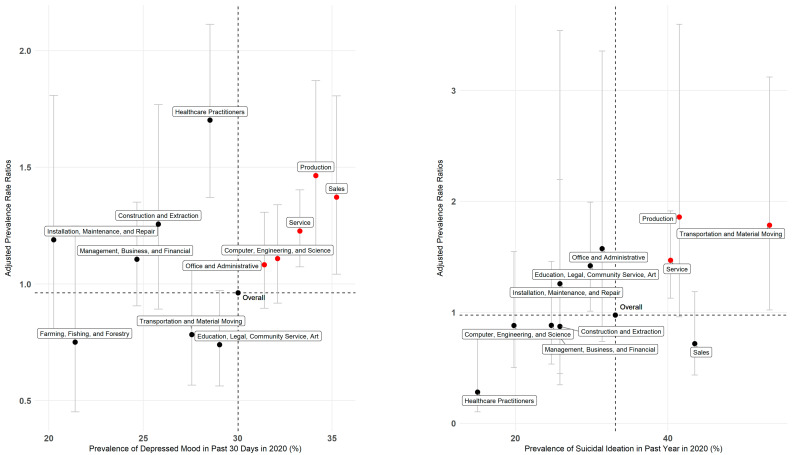
Prevalence in 2020 and adjusted rate ratios for depressed mood among California workers and suicidal ideation in the past year among California workers who have ever thought of suicide.

In 2020, 30% (CI: 28.9–31.2) of workers reported depressed mood, slightly higher than 27% (CI: 25.9–28.6) in 2019. In 2020, worker rates of depressed mood were 4% lower than expected after adjusting for prior trends (RR: 0.96, CI: 0.9–1.1) (Table 2). For depressed mood, sales, production, service, computer engineering and science, and office and administrative support were identified as priority occupation groups (Figure 1). Over a third of sales (35%, CI: 29.9–40.8) and production (34%, CI:27.1–41.7) workers reported depressed mood in 2020, with rates that were 37% (RR: 1.37, CI: 1–1.8) and 46% (RR: 1.46, CI: 1.1–1.9) higher after adjusting for prior trends, respectively. While healthcare practitioners did not meet our priority group definition due to a lower-than-average prevalence in 2020, healthcare practitioners experienced the greatest relative increase in depressed mood among all the occupation groups in 2020 (RR: 1.7, CI: 1.4–2.1) (Table 2).

Installation, maintenance, and repair occupations met the priority occupation criteria when depressed mood was defined as feeling depressed all or most of the time (2020 prevalence: 2.7%, CI: 0.6–7.4; RR: 3.09, CI: 0.9–10.5). Depressed mood all or most of the time among workers was relatively unchanged from 2019 to 2020 (see Supplemental Materials Table S2 and S3). From 2019 to 2020, absolute increases in depressed mood for all workers were driven by responses of feeling depressed some or a little of the time (+3.0%, CI: 1.0–5.0) rather than all or most of the time (−0.3%, CI: −0.9–0.4) (see Supplemental Materials Table S2).

In 2020, workers reporting a disability had the highest prevalence of depressed mood, ranging from 66% (CI: 56.0–74.8) among workers having difficulty running errands to over 70% (CI: 65.1–74.6) among workers with difficulties concentrating (Table 2). Notably, workers aged 18–29 years old had the highest prevalence of depressed mood in 2020 (43%, CI: 40.2–45.8) out of all the age groups, with the prevalence steadily increasing since 2013 (see Supplemental Figure S1). 

### 3.3. Suicidal Ideation

In 2020, 13% (CI: 11.9–13.6) of workers reported ever seriously thinking about suicide. Of those who ever seriously thought of suicide, 33% (CI: 29.4–37.0) in 2020 had seriously thought of committing suicide in the past year, a 2% increase from 2019 (31%, CI: 27.7–35.3). The suicidal ideation results among all workers can be found in the Supplemental Materials Table S7. For suicidal ideation in the past year, the priority occupation groups were the service (40%, CI: 32.3–48.8; RR: 1.47, 1.1–1.9), production (41%, CI: 20.7–64.7; RR: 1.86, CI: 1.0–3.6), and transportation and material moving occupations (53%, CI: 30.8–74.9; RR: 1.79, CI: 1.0–3.1) (Figure 1).

Workers reporting a disability had some of the highest prevalence of suicidal ideation in the past year, ranging from 49% (CI: 42.0–56.7) among workers who have difficulty concentrating to 60% (CI: 31.1–84.4) among workers having difficulty dressing or bathing. Younger workers, ages 18–29, also had a high prevalence of suicidal ideation in the past year in 2020, at 48% (CI: 42.3–53.9).

### 3.4. Other High-Risk Worker Groups

In 2020, we observed disparities in the prevalence rates of depressed mood among workers by gender and income. A greater percentage of women (33%, CI: 31.5–34.9) than men (27%, CI: 25.6–28.9) reported depressed mood. Low-income workers (incomes between $20 K–$39,999) had the highest prevalence of depressed mood (40%, CI: 36.0–43.8) compared to the other income groups. Older workers also reported high rates of adverse mental health outcomes. From 2019 to 2020, workers ages 45–65 years experienced over a 30% relative increase in both suicidal ideation in the past year (RR: 1.31 CI: 1.0–1.8) and depressed mood (RR: 1.39, CI: 1.3–1.5).

**Figure 2:**
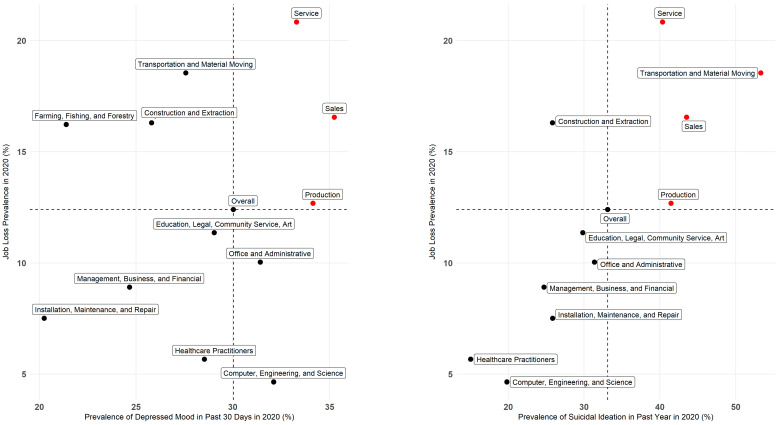
Prevalence in 2020 of job loss and depressed mood among California workers, and prevalence in 2020 of suicidal ideation in the past year among California workers who had ever thought of suicide.

In 2020, 12% (CI: 11.5–13.3) of workers reported losing their job due to the COVID-19 pandemic (Table 3). Notably, workers reporting job loss had at least a 10% absolute higher prevalence of depressed mood (39%, CI: 34.8–42.6) and suicidal ideation in the past year (45%, CI: 34.7–55.3) compared to those who did not lose their jobs (29%, CI: 27.6–30.1 and 31%, CI: 27.0–35.4, respectively). Job loss by occupation group data is available in Figure 2. Detailed results by other demographic categories for the outcomes are available in the Supplemental Materials Tables S1–S7.

## 4. Discussion

In this study, we assessed changes in mental health outcomes among California workers associated with the COVID-19 pandemic during 2020. We found sales and production workers to be among those occupations with a high prevalence and large increases in prevalent depressed mood during the pandemic; production workers were also a priority occupation group for suicidal ideation in the past year. In addition, healthcare workers had the highest relative increase in depressed mood. We also found that workers with disabilities had a high prevalence of depressed mood and suicidal ideation in the past year. A strength of our study was that we evaluated the frequency of depressed mood. Our sensitivity analysis revealed that absolute increases in depressed mood among workers were driven by less-frequent depressed mood rather than feeling depressed all or most of the time.

Similar to Czeisler et al. [1], we found disproportionate mental health outcomes among workers who were likely essential and working in person; however, our findings built upon their work by disaggregating essential workers into occupation groups, highlighting sales, production, and service workers as higher-risk. Sales workers, such as cashiers and retail salespersons, often are required to interact with the public, which could put them at increased risk for contracting COVID-19. A survey from the University of Massachusetts Labor Center found that 67% of grocery store and retail workers felt unsafe at work during the COVID-19 pandemic; many respondents noted lack of training on the prevention of SARS-CoV-2 transmission and an inability to socially distance [22]. Fear of outbreaks and death from COVID-19 may have contributed to the high rates of poor mental health among the production workers that we observed. Many production settings, including meat processing and apparel manufacturing facilities, were frequent sites of workplace outbreaks in California [23]. Production occupations also had some of the highest age-adjusted COVID-19 mortality rates in 2020 [24]. Our findings were similar to studies on healthcare workers experiencing poor mental health during the pandemic. Prasad et al. found that roughly 38% of healthcare workers reported anxiety or depression [11], which is higher than but comparable to our estimate of 29% reporting depressed mood in 2020. Moreover, work organization was affected by SARS-CoV-2 exposure and COVID-19 illness [25]. Onsite workers could have had higher demands placed on them due to lack of staff. Remote workers may have had higher demands if they had no space to work or needed to manage childcare.

Our analysis suggests that workers with disabilities may also have experienced pandemic-related mental health impacts. Using data from the 2018 Behavioral Risk Factor Surveillance System, Cree et al. found 26.6% of employed workers with any disability reported frequent mental distress [16]. The finding is considerably lower than our 2020 estimate of 59% of workers with any disability reporting depressed mood, suggesting that mental health among workers with disabilities may have worsened during the pandemic. Our higher estimate of depressed mood may be due to differing outcomes (distress vs. depressed mood) or frequency definitions, as Cree et al. examined frequent mental distress, whereas we included feeling depressed all, most, some, or a little of the time. However, when depressed mood was defined as feeling depressed all or most of the time, our estimate of workers with any disability with depressed mood was much closer to Cree’s estimate, at 10% (CI:7.5–12.9). Our findings also suggested a greater pandemic-related mental health impact on younger workers and women. These findings aligned with Panchal et al., who found a larger percentage of young adults and women reporting symptoms of depressive disorder during the pandemic compared to other age groups and men [13]. There have been documented increases in depression and anxiety among older persons (65+), especially among those with poor self-rated health [26], which may be driving the increases in depressed mood that we saw among workers ages 45–64 years.

Job loss can result in a loss of household economic stability, health insurance, and community, all of which may have increased depressed mood and suicidal ideation among workers during the pandemic. Sales, service, and production workers in our analysis were found to have a high prevalence and large increases in depressed mood and suicidal ideation as well as higher-than-average rates of job loss in 2020 due to the pandemic. These findings aligned with Panchal et al.’s finding that adults experiencing household job loss were more likely to report symptoms of depressive disorder compared to adults who did not, suggesting that job loss may be one contributing factor for the increases in depressed mood and suicidal ideation in these occupation groups during the pandemic [13].

There were several limitations to our study. First, since occupation data were only available at the major group level, we were unable to assess changes in mental health outcomes for detailed occupations. Occupation groups encompass heterogeneous working conditions, and the prevalence of depressed mood or suicidal ideation within a given occupation group may vary by detailed occupations. Second, CHIS was designed to be representative of the household population in California and not workers; thus, representativeness by occupation group is not guaranteed. Third, CHIS was not designed to follow individuals and cannot establish the temporal relationship between working and depressed mood or suicide ideation. Individuals with severe depressed mood or suicidal ideation may have left the workforce, which could explain our findings of greater increases in less frequent depressed mood compared to more frequent depressed mood. Fourth, we used a single question to evaluate depressed mood, which only considered feeling depressed in the past 30 days and may not adequately capture the depressed mood of California workers in 2020. Fifth, mental health outcomes were likely underreported given the stigma of mental illness in the United States [27], suggesting that the prevalence of adverse mental health may be higher than estimated. For example, persons with suicidal thoughts may be unlikely to disclose suicidal ideation due to stigma [28]. Few people reported suicidal ideation, resulting in an inability to detect change over time. Sixth, the CHIS methodology changed in 2019 to incorporate web-based responses, which may have increased people’s willingness to report mental health concerns compared to reporting via telephone response. Lastly, the reasons for depression and suicidal ideation remain unclear and were not ascertained in CHIS. While job loss may be one reason for adverse mental health, the causes of depressed mood and suicidal ideation among workers are multi-faceted and complex.

The ongoing COVID-19 pandemic threatens the mental health of workers and demonstrates the need to preserve and improve the mental health of the workforce. Employers in these priority occupation groups should consider mental health interventions including education, resources, and employee assistance programs [29]. Education to recognize the signs or symptoms of depression and suicidal ideation can enable employees to seek help early, and resilience training can help workers cope with pandemic-related stress. Employee assistance programs that provide free counseling and provider referrals may help workers access needed mental health services. Workplace interventions can be integrated with policy solutions, including flexible scheduling, adequate paid leave, expanded access to telemental health services, and comprehensive insurance coverage for behavioral health conditions [30].

## 5. Conclusions

California workers have experienced an increase in depressed mood and suicidal ideation since the start of the COVID-19 pandemic. Our findings suggested that workers in sales, service, production, and healthcare occupations experienced the greatest increases in adverse mental health symptoms. There is an urgent need for public health agencies and employers to provide education and resources to these worker groups to create a more resilient workforce, addressing both the immediate and long-term effects of the COVID-19 pandemic on mental health.

## Figures and Tables

**Table 1:** Sociodemographic characteristics of study population.

Study Population	2020	
	Workers	Nonworkers
	Weighted % (CI)	Weighted % (CI)
**Unweighted N**	12,982	8967
**Weighted N (CI), millions**	19.7 (19, 20)	10 (10, 10)
**Age, years**		
18–29	22.5 (22, 23)	18.7 (17.6, 19.8)
30–44	33.9 (33.4, 34.5)	10.7 (9.7, 11.7)
45–64	36.8 (36.2, 37.5)	21.7 (20.6, 22.8)
65+	6.7 (6.3, 7.1)	48.9 (47.6, 50.2)
**Sex**		
Male	52.8 (52.2, 53.4)	41.8 (40.6, 43)
Female	47.2 (46.6, 47.8)	58.2 (57, 59.4)
**Race/Ethnicity**		
Hispanic/Latino	41.8 (41, 42.5)	34.3 (32.8, 35.8)
White, NH	35.6 (35, 36.1)	43.9 (42.7, 45.2)
Black, NH	5.2 (4.8, 5.6)	6.1 (5.3, 6.8)
Asian, NH	13.7 (13.2, 14.2)	12.5 (11.5, 13.6)
AI/AN, NH/PI, 2+, NH	3.8 (3.5, 4.1)	3.1 (2.5, 3.7)
**Annual household income, $**		
<20,000	9.4 (8.5, 10.2)	20.1 (18.7, 21.5)
20,000–39,999	13.7 (12.7, 14.8)	22.4 (20.8, 24)
40,000–74,999	21.7 (20.5, 22.9)	22.6 (21.4, 23.8)
75,000–99,999	12.5 (11.6, 13.4)	10.5 (9.5, 11.6)
100,000–129,999	13.5 (12.6, 14.4)	9.9 (8.9, 11)
130,000+	29.2 (28.3, 30)	14.5 (13.4, 15.5)
**Disability**		
Blind/deaf or has severe vision/hearing problem	3.4 (2.9, 4)	10.7 (9.7, 11.7)
Difficulty concentrating (2019–2020 only)	7.4 (6.8, 8.1)	12.4 (11.2, 13.6)
Difficulty dressing or bathing (2019–2020 only)	0.6 (0.4, 0.9)	5.5 (4.6, 6.3)
Difficulty doing errands alone (2019–2020 only)	2.6 (2.1, 3)	11.2 (10.2, 12.3)
Any of the above disabilities	11.6 (10.7, 12.5)	26.6 (25.1, 28.1)
**Main Occupation (2010 Census Codes)**		
Management, business, and financial	14.4 (13.5, 15.3)	
Computer, engineering, and science	10.6 (9.9, 11.3)	
Education, legal, community service, arts, and media	13.3 (12.5, 14)	
Healthcare practitioners and technical	5.1 (4.6, 5.6)	
Service	16.6 (15.3, 17.8)	
Sales and related	7.6 (6.9, 8.2)	
Office and administrative support	11.9 (11.1, 12.7)	
Farming, fishing, and forestry	0.9 (0.6, 1.2)	
Construction and extraction	3.7 (3, 4.3)	
Installation, maintenance, and repair	2.5 (1.9, 3)	
Production	4.8 (4.1, 5.5)	
Transportation and material moving	5.5 (4.9, 6.1)	

Footnotes: Difficulty concentrating, dressing/bathing, and doing errands were not available between 2013–2018. Among workers, 2.6% (2.2, 2.9) of open-ended occupation information could not be census occupation coded. NH refers to Non-Hispanic. No occupation group results for non-workers.

**Table 2:** Depressed mood and suicidal ideation in working adults.

	Depressed Mood All, Most, Some, or a Little of the Time in the Past 30 Days				Suicidal Ideation in the Past Year among Workers Who Have Ever Thought of Suicide			
	Prevalence in 2019, % (95% CI)	Absolute Change in Prevalence from 2019 to 2020, % (95% CI)	Trend Adjusted Prevalence Ratio, PR (CI)	Model Type	Prevalence in 2019, % (95% CI)	Absolute Change in Prevalence from 2019 to 2020, % (95% CI)	Trend Adjusted Prevalence Ratio, PR (CI)	Model Type
**All Workers**	27.23 (25.9, 28.6)	2.80 (1.1, 4.5)	0.96 (0.9, 1.1)	^†††^	31.41 (27.7, 35.3)	1.71 (−3.5, 7.0)	0.98 (0.8, 1.2)	^††^
**Age**								
18–29	37.59 (34.2, 41.1)	5.40 (1.1, 9.8)	1.06 (0.9, 1.2)	^††^	38.44 (31.1, 46.2)	9.62 (0.2, 19.0)	1.08 (0.9, 1.3)	^††^
30–44	29.77 (27.8, 31.8)	−0.04 (−2.8, 2.7)	0.81 (0.7, 0.9)	^†††^	29.94 (24.1, 36.3)	−4.37 (−11.8, 3.1)	0.82 (0.6, 1.1)	^††^
45–64	19.78 (18.2, 21.5)	4.86 (2.5, 7.2)	1.39 (1.3, 1.5)	^†^	23.75 (19.7, 28.2)	−0.78 (−7.9, 6.3)	1.31 (1.0, 1.8)	^†^
65+	16.22 (13.3, 19.5)	1.40 (−3.0, 5.8)	1.09 (0.8, 1.5)	^††^	12.15 (6.4, 20.3)	7.41 (−5.0, 19.8)	1.65 (0.8, 3.5)	^†^
**Sex**								
Male	24.42 (22.7, 26.2)	2.80 (0.4, 5.2)	1.09 (1.0, 1.2)	^††^	31.83 (26.4, 37.7)	3.69 (−4.5, 11.9)	1.03 (0.8, 1.3)	^††^
Female	30.47 (28.6, 32.4)	2.70 (0.2, 5.2)	0.91 (0.8, 1.1)	^†††^	31.01 (26.3, 36.1)	−0.29 (−7.1, 6.5)	0.92 (0.7, 1.2)	^††^
**Race/Ethnicity**								
Hispanic/Latino	27.78 (25.4, 30.3)	4.48 (1.2, 7.7)	1.00 (0.9, 1.2)	^†††^	35.03 (28.3, 42.3)	5.37 (−4.1, 14.8)	1.14 (0.9, 1.5)	^††^
White, NH	25.03 (23.5, 26.6)	3.04 (0.9, 5.1)	0.97 (0.8, 1.2)	^†††^	28.45 (24.4, 32.8)	−0.48 (−6.7, 5.7)	0.95 (0.7, 1.2)	^††^
Black, NH	23.78 (19.4, 28.6)	−1.28 (−7.9, 5.3)	0.88 (0.6, 1.2)	^††^	25.23 (12.8, 41.5)	10.55 (−15.8, 36.9)	1.31 (0.6, 2.6)	^†^
Asian, NH	31.78 (28.7, 35.0)	−0.71 (−4.9, 3.5)	0.99 (0.8, 1.2)	^††^	45.64 (25.4, 56.2)	−16.09 (−30.5, −1.7)	0.52 (0.3, 0.8)	^††^
AI/AN, NH/PI, 2+, NH	29.68 (22.9, 37.2)	0.59 (−9.0, 10.2)	0.91 (0.7, 1.3)	^††^	14.41 (5.3, 29.3)	12.51 (−4.5, 29.6)	1.47 (0.8, 2.8)	^†^
**Annual household income**								
<20,000	36.06 (31.3, 41.0)	2.53 (−4.1, 9.1)	1.05 (0.9, 1.3)	^††^	48.62 (33.1, 64.3)	−5.69 (−25.5, 14.1)	0.82 (0.5, 1.3)	^††^
20,000–39,999	36.58 (33.0, 40.3)	3.25 (−2.0, 8.5)	0.93 (0.7, 1.2)	^†††^	38.41 (28.6, 48.9)	4.85 (−8.5, 18.2)	1.48 (1.1, 1.9)	^†^
40,000–74,999	27.59 (25.0, 30.3)	3.80 (0.1, 7.5)	1.01 (0.9, 1.2)	^††^	31.28 (23.1, 40.4)	7.61 (−3.9, 19.1)	1.11 (0.8, 1.6)	^††^
75,000–99,999	25.45 (21.9, 29.3)	2.51 (−2.2, 7.2)	1.11 (0.9, 1.4)	^††^	31.43 (22.1, 42.0)	−2.56, (−16.1, 11.0)	1.27 (0.8, 1.9)	^†^
100,000–129,999	25.73 (22.5, 29.2)	2.63 (−1.8, 7.0)	1.04 (0.9, 1.3)	^††^	33.57 (23.8, 44.5)	−9.95 (−22.5, 2.5)	0.68 (0.4, 1.1)	^††^
130,000+	20.48 (18.5, 22.6)	2.84 (0.1, 5.5)	1.21 (1.0, 1.4)	^††^	18.86 (13.8, 24.8)	6.15 (−2.5, 14.8)	2.14 (0.9, 5.2)	^†††^
**Disability**								
Blind/deaf or has severe vision/hearing problem								
Yes	37.91 (30.2, 46.1)	1.23 (−10.8, 13.3)	0.96 (0.7, 1.3)	^††^	47.53 (29.1, 66.5)	−27.91 (−51.3, −4.5)	0.58 (0.3, 1.3)	^†^
No	26.88 (25.5, 28.2)	2.82 (1.1, 4.6)	0.95 (0.9, 1.1)	^†††^	30.90 (27.3, 34.7)	2.65 (−2.6, 7.9)	1.02 (0.9, 1.2)	^††^
Difficulty concentrating (2019 and later)								
Yes	71.32 (64.8, 77.3)	−1.30 (−8.9, 6.3)	…	…	50.87 (41.9, 59.8)	−1.53 (−12.8, 9.7)	…	…
No	24.34 (23.0, 25.7)	2.47 (0.7, 4.2)	…	…	27.26 (23.3, 31.5)	0.40 (−5.4, 6.2)	…	…
Difficulty dressing or bathing (2019 and later)								
Yes	61.68 (42.4, 78.6)	4.37 (−19.0, 27.8)	…	…	57.81 (33.1, 80.0)	2.08 (−32.8, 36.9)	…	…
No	26.94 (25.6, 28.3)	2.86 (1.1, 4.6)	…	…	30.83 (27.1, 34.7)	1.62 (−3.6, 6.8)	…	…
Difficulty doing errands alone (2019 and later)								
Yes	61.86 (52.8, 70.4)	3.95 (−8.4, 16.3)	…	…	50.26 (38.5, 62.0)	1.55 (−15.2, 18.3)	…	…
No	26.26 (24.9, 27.6)	2.84 (1.1, 4.6)	…	…	30.05 (26.2, 34.1)	1.26 (−4.3, 6.8)	…	…
Any of the above disabilities	57.53 (52.5, 62.4)	1.42 (−4.8, 7.6)	…	…	50.48 (42.1, 58.8)	−5.20 (−16.0, 5.7)	…	…
**Main Occupation (2010 Census Codes)**								
Management, business, and financial	25.39 (22.6, 28.4)	0.72 (−4.6, 3.1)	1.11 (0.9, 1.4)	^††^	27.68 (19.6, 37.0)	−2.95 (−14.7, 8.8)	0.88 (0.5, 1.5)	^††^
Computer, engineering, and science	28.47 (24.9, 32.3)	3.65 (−1.0, 8.3)	1.11 (0.9, 1.3)	^††^	26.16 (16.8, 37.4)	−6.34 (−18.6, 6.0)	0.88 (0.5, 1.5)	^†^
Education, legal, community service, arts, and media	29.08 (25.8, 32.5)	−0.04 (−4.4, 4.4)	0.74 (0.6, 1.0)	^†††^	21.87 (15.2, 29.8)	7.95 (−2.5, 18.4)	1.42 (1.0, 2.0)	^†^
Healthcare practitioners and technical	21.98 (18.2, 26.1)	6.56 (0.9, 12.3)	1.70 (1.4, 2.1)	^†^	36.97 (20.5, 56.0)	−21.91 (−42.2, −1.6)	0.28 (0.1, 0.8)	^††^
Service	31.65 (28.1, 35.3)	1.64 (−3.6, 6.9)	1.23 (1.1, 1.4)	^†^	41.15 (29.9, 53.1)	−0.80 (−14.5, 12.9)	1.47 (1.1, 1.9)	^†^
Sales and related	24.15 (20.0, 28.7)	11.10 (4.3, 17.8)	1.37 (1.0, 1.8)	^††^	52.84 (37.7, 67.6)	−9.32 (−28.7, 10.1)	0.72 (0.4, 1.2)	^††^
Office and administrative support	27.47 (23.8, 31.4)	3.95 (−1.0, 8.9)	1.08 (0.9, 1.3)	^††^	31.69 (22.5, 42.1)	−0.31 (−13.5, 12.9)	1.57 (0.7, 3.4)	^†††^
Farming, fishing, and forestry *	18.86 (9.6, 31.6)	2.53 (−12.0, 17.1)	0.75 (0.5, 1.2)	^†^	…	…	…	^†^
Construction and extraction	24.22 (15.6, 34.7)	1.59 (−10.4, 13.6)	1.26 (0.9, 1.8)	^†^	38.48 (10.3, 74.0)	−12.63 (−52.2, 27.0)	0.87 (0.3, 2.2)	^†^
Installation, maintenance, and repair	17.69 (9.8, 28.3)	2.57 (−8.7, 13.8)	1.19 (0.8, 1.8)	^†^	12.90 (1.1, 43.1)	12.96 (−18.0, 43.9)	1.26 (0.4, 3.5)	^†^
Production	19.70 (14.3, 26.1)	14.44 (5.3, 23.6)	1.46 (1.1, 1.9)	^†^	37.07 (4.2, 83.9)	4.42 (−44.0, 52.8)	1.86 (1.0, 3.6)	^†^
Transportation and material moving	32.85 (24.3, 42.3)	−5.28 (−15.5, 4.9)	0.78 (0.6, 1.1)	^††^	28.80 (10.3, 54.6)	24.51 (−6.2, 55.3)	1.79 (1.0, 3.1)	^†^

Notes: ^†^ designates intercept. ^††^ designates linear. ^†††^ designates quadratic. NH refers to Non-Hispanic. * Farming, fishing, and forestry occupations had their suicidal ideation results suppressed due to small cell sizes. There are no adjusted results for difficulty concentrating, difficulty dressing or bathing, difficulty doing errands alone, and any of the above disabilities due to no data from 2013–2018. The “…” for the Farming, fishing, and forestry results is for suppression; there are no results for suicidal ideation due to small cell sizes.

**Table 3:** Job loss and reduced hours among working adults in 2020.

	Suicidal Ideation in the Past Year among Workers Who Have Ever Thought of Suicide		Depressed Mood All, Most, Some, or a Little of the Time in the Past 30 Days		All Workers	
	Prevalence in 2020 (%)	(95% CI)	Prevalence in 2020 (%)	(95% CI)	Prevalence in 2020 (%)	(95% CI)
**Job Changes**						
Lost job	44.81	(34.7, 55.3)	38.68	(34.8, 42.6)	12.40	(11.5, 13.3)
Did not lose job	31.11	(27.0, 35.4)	28.8	(27.6, 30.1)		
Reduced hours	38.10	(32.0, 44.5)	35.65	(33.0, 38.4)	24.37	(23.2, 25.6)
Did not have reduced hours	30.94	(26.5, 35.7)	28.22	(27.1, 29.4)		

## Data Availability

The California Department of Public Health does not have permission to release our funder file data. Aggregate level, public use files of CHIS data can be accessed through the ASK CHIS website.

## Supplementary Materials

The following supporting information can be downloaded at: https://www.mdpi.com/article/10.3390/ijerph20021253/s1, Table S1: Universe of Respondents to Suicidal Ideation in Past Year among Adults Who Have Ever Thought of Suicide; Table S2: Sensitivity Analysis for Depressed Mood by Demographic Categories, Working Adults; Table S3: Sensitivity Analysis for Depressed Mood by Employment Information, Working Adults; Table S4: Study Population, All Demographics, Table S5: Study Population for Individual Years, 2013–2020; Table S6: Study Population for Individual Years, Employment Information 2013–2020; Table S7: Suicidal Ideation in Working Adults; Figure S1: Depressed mood in the past 30 days from 2013 to 2020 by age category.
