# Supplementary material for: Increases in Prevalent Depressed Mood and Suicidal Ideation among Workers during the COVID-19 Pandemic—Findings from the California Health Interview Survey
Source: Int J Environ Res Public Health. 2023 Jan 10;20(2):1253. doi: 10.3390/ijerph20021253 (PMC9859206; doi:10.3390/ijerph20021253)

Supplemental Materials

Table S1. Universe of Respondents to Suicidal Ideation in Past Year

Adults Who Have Ever Thought of Suicide	Universe of Respondents to Suicidal Ideation in the Past Year (Adults Who Have Ever Thought of Suicide)	
	2020	
	Workers Weighted % (CI)	Nonworkers Weighted % (CI)
**Unweighted N**	1661	964
**Weighted N (CI), millions**	2.5 (2.3, 2.7)	1.1 (1.0, 1.2)
**Age, years**		
18-29	37.3 (34.1, 40.5)	35.3 (30.5, 40.4)
30-44	35.2 (32.1, 38.4)	13.0 (10.0, 16.0)
45-64	24.2 (21.2, 27.1)	24.3 (20.1, 28.4)
65+	3.4 (2.6, 4.2)	27.3 (23.5, 31.0)
**Sex**		
Male	50.0 (46.5, 53.5)	42.2 (37.5, 46.8)
Female	50.0 (46.5, 53.5)	57.8 (53.2, 62.5)
**Race/Ethnicity**		
Hispanic/Latino	38.8 (35.2, 42.5)	37.2 (32.1, 42.3)
White, NH	44.3 (41.0, 47.7)	45.6 (40.9)
Black, NH	3.0 (1.8, 4.2)	4.5 (2.4, 6.5)
Asian, NH	9.1 (7.4, 10.8)	7.4 (5.3, 9.5)
AI/AN, NH/PI, 2+, NH	4.8 (3.6, 6.0)	5.4 (2.7, 8.1)
**Highest Education**		
Less than High School	3.8 (1.9, 5.6)	10.8 (7.1, 14.5)
Grade 12/ High School Diploma	19.9 (16.6, 23.2)	27.0 (21.7, 32.2)
Some College or AA Degree	28.1 (25.0, 31.2)	32.8 (28.0, 37.5)
BA or BS Degree/Some Graduate School	28.2 (25.2, 31.1)	19.5 (16.3, 22.6)
Masters Degree or Higher	20.1 (17.3, 22.8)	10.1 (7.8, 12.3)
**Family Poverty Threshold Level**		
0-138%	14.6 (12.5, 16.7)	31.6 (26.2, 37.0)
139-249%	16.4 (13.8, 19.0)	21.3 (17.0, 25.5)
250-399%	17.9 (15.0, 20.7)	18.3 (14.2, 22.5)
400%+	51.2 (47.7, 54.6)	28.8 (25.0, 32.6)
**Annual household income, $**		
<20,000	8.7 (6.9, 10.5)	20.7 (16.8, 24.7)
20,000-39,999	17.7 (15.1, 20.4)	24.2 (19.5, 29.0)
40,000-74,999	21.5 (18.4, 24.6)	26.2 (21.8, 30.6)
75,000-99,999	13.2 (10.8, 15.5)	8.5 (6.0, 11.0)
100,000-129,999	13.0 (10.8, 15.1)	10.3 (6.4, 14.1)
130,000+	25.9 (23.3, 28.5)	10.1 (7.7, 12.5)
**Disability**		
Blind/Deaf or Has Severe Vision/Hearing Problem	3.1 (1.7, 4.5)	8.1 (5.3, 10.9)
Difficulty Concentrating (2019-2020 only)	25.2 (22.3, 28.1)	37.5 (32.8, 42.1)
Difficulty Dressing or Bathing (2019-2020 only)	2.5 (1.1, 3.8)	6.0 (3.9, 8.1)
Difficulty Doing Errands Alone (2019-2020 only)	8.8 (6.9, 10.8)	20.7 (16.3, 25.2)
Any of the above disabilities	29.7 (26.6, 32.9)	51.1 (46.3, 55.9)
**Sexual Orientation (did not ask adults >70 y/o in 2013-2014)**		
Heterosexual	77.5 (74.9, 80.1)	77.6 (73.6, 81.6)
Not heterosexual	22.5 (19.9, 25.1)	22.4 (18.4, 26.4)
**Gender**		
Cisgender	96.5 (95.3, 97.7)	99.0 (98.0, 99.9)
Transgender or gender non-conforming	3.5 (2.3, 4.7)	1.0 (0.1, 2.0)
**Employer Type at Main Job**		
Private	69.6 (66.0, 73.1)	. .
Government	18.6 (15.8, 21.4)	. .
Self-employed	10.0 (8.1)	. .
Family business or farm	1.8 (0.9, 2.7)	. .
**Main Industry (2010 Census Codes)**		
Agriculture, Forestry, Fishing, Hunting and Mining	0.6 (0.1, 1.2)	. .
Construction	3.4 (2.0, 4.9)	. .
Manufacturing	6.9 (4.9, 8.9)	. .
Wholesale Trade	0.5 (0.1, 0.9)	. .
Retail Trade	9.9 (7.9, 12.0)	. .
Transportation, Warehousing, and Utilities	3.0 (1.7, 4.3)	. .
Information	4.0 (2.6, 5.5)	. .
Finance and Insurance, Real Estate, Rental and Leasing	6.2 (4.7, 7.7)	. .
Professional, Scientific, Management	14.4 (12.2, 16.7)	. .
Educational Services, Health Care and Social Assistance	28.0 (25.1, 30.8)	. .
Arts, Entertainment, Recreation, Accommodation, and Food Services	10.0 (7.6, 12.4)	. .
Other Services, Except Public Administration	4.0 (2.6, 5.3)	. .
Public Administration	5.5 (4.3, 6.7)	. .
**Main Occupation (2010 Census Codes)**		
Management, Business, and Financial	12.0 (9.8, 14.1)	. .
Computer, Engineering, and Science	10.8 (8.9, 12.6)	. .
Education, Legal, Community Service, Arts, and Media	19.3 (16.8, 21.8)	. .
Healthcare Practitioners and Technical	5.7 (4.5, 6.8)	. .
Service	16.1 (13.5, 18.7)	. .
Sales and Related	8.9 (6.7, 11.2)	. .
Office and Administrative Support	11.8 (9.8, 13.7)	. .
Farming, Fishing, and Forestry	0.4 (-0.1, 1.0)	. .
Construction and Extraction	2.8 (1.3, 4.3)	. .
Installation, Maintenance, and Repair	2.2 (0.9, 3.4)	. .
Production	3.8 (2.2, 5.5)	. .
Transportation and Material Moving	4.0 (2.3, 5.6)	. .

Footnotes:

Among workers who had ever seriously thought about suicide, 1.5% (0.9, 2.1) of open-ended occupation information could not be census occupation coded.

Among workers who had ever seriously thought of suicide, 2.2% (1.6, 2.8) of open-ended industry information could not be census occupation coded.

Employer type asks respondents whether they were employed by a private company, the government, self-employed, family business/farm.

Sexual Orientation was coded as heterosexual vs. non-heterosexual by collapsing the “gay, lesbian, or homosexual”, “bisexual,” “not sexual/celibate/ none,” and “other” categories into non-heterosexual

NH refers to Non-Hispanic

Table S2. Sensitivity Analysis for Depressed Mood by Demographic Categories, Working Adults

	Depressed Mood All, Most, Some, or a Little of the Time in the Past 30 Days				Depressed Mood All, or Most of the Time in the Past 30 Days				Depressed Mood Some or A Little of the Time in the Past 30 Days			
	Prevalence in 2019, % (95% CI)	Absolute Change in Prevalence from 2019 to 2020, % (95% CI)	Trend Adjusted Prevalence Ratio, PR (CI)	Model Type	Prevalence in 2019, % (95 % CI)	Absolute Change in Prevalence from 2019 to 2020, % (95% CI)	Trend Adjusted Prevalence Ratio, PR (CI)	Model Type	Prevalence in 2019, % (CI)	Absolute Change in Prevalence from 2019 to 2020, % (95% CI)	Trend Adjusted Prevalence Ratio, PR (CI)	Model Type
**All Workers**	27.23 (25.9, 28.6)	2.80 (1.1, 4.5)	0.96 (0.9, 1.1)	†††	2.81 (2.4, 3.3)	-0.28 (-0.9-0.4)	0.91 (0.7, 1.2)	††	24.41 (23.1, 25.7)	3.08 (1.3-4.8)	0.98 (0.9, 1.1)	†††
**Age**												
18-29	37.59 (34.2, 41.1)	5.40 (1.1, 9.8)	1.06 (0.9, 1.2)	††	5.12 (3.7, 6.9)	0.24 (-1.9, 2.4)	0.97 (0.6, 1.5)	††	32.47 (29.2, 35.8)	5.17 (1.0, 9.3)	1.07 (0.9, 1.2)	††
30-44	29.77 (27.8, 31.8)	-0.04 (-2.8, 2.7)	0.81 (0.7, 0.9)	†††	2.82 (2.2, 3.6)	-0.73 (-1.7, 0.3)	0.73 (0.4, 1.2)	††	26.95 (25.0, 28.9)	0.68 (-1.9, 3.3)	0.82 (0.7, 1.0)	†††
45-64	19.78 (18.2, 21.5)	4.86 (2.5, 7.2)	1.39 (1.3, 1.5)	†	1.57 (1.2, 2.1)	-0.27 (-0.9, 0.4)	1.01 (0.7, 1.5)	†	18.21 (16.6, 19.9)	5.13 (2.6, 7.6)	1.42 (1.3, 1.6)	†
65+	16.22 (13.3, 19.5)	1.40 (-3.0, 5.8)	1.09 (0.8, 1.5)	††	1.01 (0.4, 2.1)	1.08 (-0.4, 2.6)	1.53 (0.4, 5.4)	†	15.20 (12.3, 18.4)	0.33 (-3.9, 4.5)	1.03 (0.8, 1.4)	††
**Sex**												
Male	24.42 (22.7, 26.2)	2.80 (0.4, 5.2)	1.09 (1.0, 1.2)	††	2.53 (2.0, 3.2)	-0.07 (-1.0, 0.8)	0.88 (0.6, 1.3)	††	21.89 (20.2, 23.7)	2.87 (0.5, 5.2)	1.11 (1.0, 1.2)	††
Female	30.47 (28.6, 32.4)	2.70 (0.2, 5.2)	0.91 (0.8, 1.1)	†††	3.14 (2.4, 4.0)	-0.52 (-1.5, 0.5)	0.94 (0.6, 1.4)	††	27.33 (25.5, 29.2)	3.22 (0.7, 5.7)	0.93 (0.8, 1.1)	†††
**Race/Ethnicity**												
Hispanic/Latino	27.78 (25.4, 30.3)	4.48 (1.2, 7.7)	1.00 (0.9, 1.2)	†††	3.34 (2.5, 4.3)	-0.37 (-1.6, 0.8)	1.38 (1.0, 1.9)	†	24.44 (22.2, 26.8)	4.85 (1.7, 8.0)	1.18 (1.0, 1.3)	††
White, NH	25.03 (23.5, 26.6)	3.04 (0.9, 5.1)	0.97 (0.8, 1.2)	†††	2.07 (1.7, 2.6)	-0.13 (-0.7, 0.5)	0.84 (0.6, 1.2)	††	22.96 (21.5, 24.5)	3.17 (1.1, 5.2)	0.97, 0.8, 1.1	†††
Black, NH	23.78 (19.4, 28.6)	-1.28 (-7.9, 5.3)	0.88 (0.6, 1.2)	††	4.33 (1.7, 8.9)	-2.97 (-6.4, 0.5)	0.58 (0.2, 1.7)	†	19.45 (15.1, 24.4)	1.69 (-4.9, 8.3)	1.00 (0.7, 1.4)	††
Asian, NH	31.78 (28.7, 35.0)	-0.71 (-4.9, 3.5)	0.99 (0.8, 1.2)	††	2.64 (1.4, 4.5)	0.21 (-1.6, 2.0)	0.92 (0.4, 1.9)	††	29.13 (26.3, 32.1)	-0.92, (-4.9, 3.1)	1.00 (0.8, 1.2)	††
AI/AN, NH/PI, 2+, NH	29.68 (22.9, 37.2)	0.59 (-9.0, 10.2)	0.91 (0.7, 1.3)	††	2.56 (0.9, 5.6)	1.21 (-3.0, 5.4)	0.83 (0.2, 3.8)	††	27.13 (20.8, 34.3)	-0.62 (-9.6, 8.3)	0.92 (0.6, 1.3)	††
**Highest Education**												
Less than High School	28.33 (22.2, 35.1)	2.99 (-5.0, 11.0)	1.00 (0.8, 1.2)	†	2.42 (1.0, 4.9)	-0.56 (-2.9, 1.7)	0.68 (0.3, 1.8)	†	25.90 (20.2, 32.2)	3.56 (-4.1, 11.2)	1.03 (0.8, 1.3)	†
Grade 12/ High School Diploma	24.45 (20.9, 28.3)	4.30 (-0.5, 9.1)	1.17 (1.0, 1.4)	††	3.83 (2.5, 5.6)	-0.50 (-2.5, 1.5)	0.84 (0.5, 1.5)	††	20.62 (17.6, 23.8)	4.79 (0.5, 9.1)	1.3 (1.2, 1.5)	†
Some College or AA Degree	31.46 (28.8, 34.2)	1.43 (-2.1, 4.9)	0.83 (0.7, 1.0)	†††	3.90 (2.8, 5.3)	-0.31 (-1.9, 1.3)	0.85 (0.5, 1.3)	††	27.56 (24.9, 30.4)	1.74 (-1.8, 5.3)	0.82 (0.7, 1.0)	†††
BA or BS Degree/Some Graduate School	28.31 (26.6, 30.0)	2.16 (-0.6, 4.9)	0.92 (0.8, 1.1)	†††	2.27 (1.7, 3.0)	-0.35 (-1.2, 0.5)	0.89 (0.5, 1.6)	††	26.05 (24.3, 27.9)	2.51 (-0.2, 5.2)	1.15 (1.0, 1.3)	††
Masters Degree or Higher	23.06 (20.9, 25.3)	3.52 (0.7, 6.4)	1.13 (0.9, 1.4)	††	1.49 (1.0, 2.1)	0.44 (-0.4, 1.3)	2.61 (1.4, 4.7)	†	21.57 (19.4, 23.8)	3.08 (0.3, 5.9)	1.11 (0.9, 1.3)	††
**Family Poverty Threshold Level**												
0-138%	36.37 (32.7, 40.2)	2.70 (-2.3, 7.7)	1.09 (0.9, 1.3)	††	4.90 (3.2, 7.1)	-0.30 (-2.7, 2.1)	0.89 (0.5, 1.5)	††	31.47 (28.1, 35.0)	3.00 (-2.1, 8.1)	1.12 (0.9,1.3)	††
139-249%	30.22 (26.2, 34.5)	5.64 (0.1, 11.2)	1.18 (1.0, 1.4)	††	3.51 (2.5, 4.9)	-0.65 (-2.2, 0.9)	0.74 (0.4, 1.3)	††	26.71 (22.8, 30.9)	6.29 (0.9, 11.7)	1.24 (1.0, 1.5)	††
250-399%	28.21 (25.2, 31.3)	-0.78 (-4.8, 3.3)	0.96 (0.8, 1.1)	††	2.88 (1.9, 4.1)	-0.45 (-2.0, 1.1)	0.42 (0.2, 1.0)	†††	25.32 (22.5, 28.3)	-0.33 (-4.2, 3.5)	0.96 (0.8, 1.1)	††
400%+	22.85 (21.4, 24.3)	3.66 (1.7, 5.7)	0.95 (0.8, 1.1)	†††	1.87 (1.4, 2.5)	0.00 (-0.7, 0.7)	1.03 (0.7, 1.6)	††	20.98 (19.7, 22.4)	3.66 (1.8, 5.5)	0.97 (0.8, 1.1)	†††
**Annual household income**												
<20,000	36.06 (31.3, 41.0)	2.53 (-4.1, 9.1)	1.05 (0.9, 1.3)	††	5.49 (3.0, 9.1)	-0.02 (-3.4, 3.4)	1.30 (0.8, 2.)	†	30.58 (26.2, 35.2)	2.55 (-4.2, 9.3)	1.20 (1.0, 1.4)	†
20,000-39,999	36.58 (33.0, 40.3)	3.25 (-2.0, 8.5)	0.93 (0.7, 1.2)	†††	4.72 (3.3, 6.5)	-1.50 (-3.6, 0.6)	0.78 (0.4, 1.4)	††	31.86 (28.3, 35.6)	4.75 (-0.5, 10.0)	1.24 (1.0, 1.5)	††
40,000-74,999	27.59 (25.0, 30.3)	3.80 (0.1, 7.5)	1.01 (0.9, 1.2)	††	2.00 (1.4, 2.8)	0.82 (-0.5, 2.1)	1.03 (0.6, 1.9)	††	25.59 (23.1, 28.2)	2.98 (-0.8, 6.7)	1.00 (0.9, 1.2)	††
75,000-99,999	25.45 (21.9, 29.3)	2.51 (-2.2, 7.2)	1.11 (0.9, 1.4)	††	3.24 (2.1, 4.8)	-0.97 (-2.7, 0.7)	0.81 (0.4, 1.8)	††	22.21 (18.9, 25.8)	3.49 (-1.0, 7.9)	1.14 (0.9, 1.4)	††
100,000-129,999	25.73 (22.5, 29.2)	2.63 (-1.8, 7.0)	1.04 (0.9, 1.3)	††	3.22 (2.1, 4.7)	-1.61 (-3.0, -0.2)	0.49 (0.2, 1.0)	††	22.51 (19.4, 25.9)	4.24 (0.0, 8.5)	1.11 (0.9, 1.4)	††
130,000+	20.48 (18.5, 22.6)	2.84 (0.1, 5.5)	1.21 (1.0, 1.4)	††	1.16 (0.7, 1.8)	0.44 (-0.3, 1.2)	1.23 (0.6, 2.4)	††	19.31 (17.4, 21.3)	2.40 (-0.1, 4.9)	1.20 (1.0, 1.4)	††
**Disability**												
Blind/Deaf or Has Severe Vision/Hearing Problem	37.91 (30.2, 46.1)	1.23 (-10.8, 13.3)	0.96 (0.7, 1.3)	††	2.09 (0.6, 5.1)	3.92 (-1.7, 9.5)	1.14 (0.4, 3.2)	†	35.82 (28.2, 44.0)	-2.70 (-14.2, 8.8)	0.94 (0.7, 1.3)	††
Not Blind/Deaf or Has Severe Vision/Hearing Problem	26.88 (25.5, 28.2)	2.82 (1.1, 4.6)	0.95 (0.9, 1.1)	†††	2.84 (2.4, 3.4)	-0.43 (-1.1, 0.2)	0.89 (0.7, 1.2)	††	24.04 (22.7, 25.4)	3.25 (1.5, 5.0)	0.99 (0.9, 1.1)	†††
Difficulty Concentrating*	71.32 (64.8, 77.3)	-1.30 (-8.9, 6.3)	. .	. .	17.84 (13.6, 22.8)	-4.38 (-10.2, 1.5)	. .	. .	53.48 (47.5, 59.4)	3.07 (-4.7, 10.9)	. .	. .
Denied Difficulty Concentrating*	24.34 (23.0, 25.7)	2.47 (0.7, 4.2)	. .	. .	1.83 (1.5, 2.2)	-0.17 (-0.7, 0.4)	. .	. .	22.51 (21.3, 23.8)	2.65 (0.9, 4.4)	. .	. .
Difficulty Dressing or Bathing*	61.68 (42.4, 78.6)	4.37 (-19.0, 27.8)	. .	. .	20.87 (7.9, 40.3)	-4.12 (-24.7, 16.4)	. .	. .	40.82 (17.0, 68.4)	8.49 (-22.9, 33.9)	. .	. .
Denied Difficulty Dressing or Bathing*	26.94 (25.6, 28.3)	2.86 (1.1, 4.6)	. .	. .	2.67 (2.2, 3.1)	-0.22 (-0.9, 0.4)	. .	. .	24.28 (23.0, 25.6)	3.08 (1.3, 4.8)	. .	. .
Difficulty Doing Errands Alone*	61.86 (52.8, 70.4)	3.95 (-8.4, 16.3)	. .	. .	17.43 (11.6, 24.6)	-3.94 (-12.1, 4.2)	. .	. .	44.43 (36.4, 52.7)	7.89 (-4.5, 20.3)	. .	. .
Denied Difficulty Doing Errands Alone*	26.26 (24.9, 27.6)	2.84 (1.1, 4.6)	. .	. .	2.40 (2.0, 2.9)	-0.16 (-0.8, 0.5)	. .	. .	23.85 (22.6, 25.2)	2.99 (1.2, 4.7)	. .	. .
Any of the above disabilities	57.53 (52.5, 62.4)	1.42 (-4.8, 7.6)	. .	. .	12.13 (9.6, 15.0)	-2.19 (-5.9, 1.5)	. .	. .	45.41 (41.0, 49.9)	3.61 (-2.6, 9.8)	. .	. .
**Sexual Orientation (did not ask adults >70 y/o in 2013-2014)**												
Heterosexual	25.74 (24.3, 27.2)	2.97 (1.1, 4.8)	0.98 (0.9, 1.1)	†††	2.64 (2.2, 3.2)	1.74 (-0.6, 4.1)	0.86 (0.6, 1.2)	††	23.10 (21.7, 24.5)	3.45 (1.6, 5.3)	1.02 (0.9, 1.2)	†††
Not heterosexual	39.77 (35.5, 44.2)	2.49 (-3.4, 8.4)	1.00 (0.9, 1.2)	††	4.24 (3.1, 5.7)	-0.48 (-1.2, 0.2)	1.18 (0.7, 2.0)	††	35.53 (31.2 (40.0)	0.75 (-5.2, 6.7)	0.98 (0.8, 1.2)	††

Notes:

Sexual orientation was dichotomized as heterosexual vs. non-heterosexual by collapsing the “gay, lesbian, or homosexual”, “bisexual,” “not sexual / celibate / none,” and “other” categories into non-heterosexual.

*Difficulty concentrating, Dressing/Bathing, and Doing errands were not available 2013-2018.

NH refers to Non-Hispanic.

Table S3. Sensitivity Analysis for Depressed Mood by Employment Information, Working Adults

	Depressed Mood All, Most, Some, or a Little of the Time in the Past 30 Days				Depressed Mood All, or Most of the Time in the Past 30 Days				Depressed Mood Some or A Little of the Time in the Past 30 Days			
	**Prevalence in 2019, % (95% CI)**	**Absolute Change in Prevalence from 2019 to 2020, % (95% CI)**	**Trend Adjusted Prevalence Ratio, PR (CI)**	**Model Type**	**Prevalence in 2019, % (95% CI)**	**Absolute Change in Prevalence from 2019 to 2020, % (95% CI)**	**Trend Adjusted Prevalence Ratio, PR (CI)**	**Model Type**	**Prevalence in 2019, % (95% CI)**	**Absolute Change in Prevalence from 2019 to 2020, % (95% CI)**	**Trend Adjusted Prevalence Ratio, PR (CI)**	**Model Type**
**Employer Type at Main Job**												
Private	27.63 (25.9, 29.4)	3.87 (1.5, 6.3)	0.98 (0.9, 1.1)	†††	2.97 (2.4, 3.6)	-0.46 (-1.3, 0.4)	0.83 (0.6, 1.1)	††	24.66 (23.0, 26.4)	4.33 (1.9, 6.7)	1.02 (0.9, 1.2)	†††
Government	26.39 (23.8, 29.1)	2.14 (-1.4, 5.7)	0.83 (0.7, 1.1)	†††	2.90 (1.7, 4.6)	-0.14 (-2.0, 1.7)	1.90 (1.0, 3.5)	†	23.49 (20.9, 26.2)	2.29 (-1.4, 5.9)	0.85 (0.7, 1.1)	†††
Self-employed	24.58 (21.7, 27.7)	-1.12 (-5.5, 3.2)	0.94 (0.8, 1.1)	††	1.99 (1.2, 3.2)	0.06 (-1.3, 1.5)	1.21 (0.7, 2.2)	†	22.59 (19.8, 25.5)	-1.18 (-5.2, 2.9)	0.94 (0.8, 1.1)	††
Family business or farm	33.16 (24.4, 42.9)	3.85 (-9.5, 17.2)	1.29 (0.9, 1.8)	†	2.85 (0.8, 6.9)	1.64 (-3.5, 6.7)	1.14 (0.3, 5.0)	†	30.30 (21.9, 39.8)	2.21 (-10.7, 15.2)	1.31 (0.9, 1.9)	†
**Main Industry (2010 Census Codes)**												
Agriculture, Forestry, Fishing, Hunting and Mining	27.94 (19.5, 37.6)	-5.11 (-19.1, 8.9)	0.90 (0.5, 1.5)	†	0.96 (0.1, 3.9)	-0.13 (-2.0, 1.7)	0.50 (0.1, 1.8)	†	26.97 (18.6, 36.7)	-4.98 (-19.0, 9.0)	0.93 (0.5, 1.6)	†
Construction	23.69 (17.1, 31.3)	2.73 (-6.7, 12.2)	1.28 (1.0, 1.7)	†	3.19 (1.2, 6.9)	-1.36 (-4.3, 1.5)	0.41 (0.1, 1.3)	††	20.50 (14.6, 27.6)	4.09 (-4.8, 13.0)	1.30 (1.0, 1.7)	†
Manufacturing	27.83 (23.2, 32.9)	4.00 (-2.5, 10.5)	1.10 (0.9, 1.4)	††	2.66 (1.1, 5.3)	-0.33 (-2.5, 1.8)	2.28 (1.2, 4.4)	†	25.17 (20.9, 29.8)	4.33 (-1.9, 10.6)	1.10 (0.8, 1.4)	††
Wholesale Trade	27.17 (15.8, 41.3)	-13.65 (-27.2, -0.2)	0.6 (1.4, 1.0)	†	..	0.56 (-0.4, 1.5)	0.29 (0.0, 11.2)	†	27.17 (15.8, 41.3)	-14.21 (-27.7, -0.7)	0.62 (0.4, 1.1)	†
Retail Trade	27.65 (23.0, 32.7)	5.38 (-1.5, 12.2)	1.06 (0.8, 1.4)	††	3.88 (2.1, 6.6)	0.22 (-2.9, 3.3)	1.83 (0.9, 3.6)	†	23.77 (19.5, 28.5)	5.16 (-1.4, 11.7)	1.05 (0.8, 1.4)	††
Transportation, Warehousing, and Utilities	20.93 (15.6, 27.1)	8.62 (1.2, 16.1)	1.54 (1.2, 1.9)	†	2.60 (1.2, 4.8)	-1.30 (-3.4,0.8)	0.82 (0.2, 3.4)	†	18.32 (13.3, 24.3)	9.91 (2.6, 17.2)	1.60 (1.3, 2.0)	†
Information	33.96 (27.2, 41.2)	-2.48 (-12.1, 7.2)	1.04 (0.7, 1.6)	††	3.07 (0.6, 8.7)	-2.28 (-5.8, 1.3)	0.14 (0.0, 0.7)	††	30.89 (25.0, 37.2)	-0.20 (-9.2, 8.8)	1.20 (0.8, 1.8)	††
Finance and Insurance, Real Estate, Rental and Leasing	22.44 (17.9, 27.5)	2.79 (-3.3, 8.8)	1.47 (1.2, 1.8)	†	2.79 (1.6, 4.6)	-1.68 (-3.2, -0.1)	1.16 (0.5, 2.7)	†	19.64 (15.1, 24.8)	4.47 (-1.7, 10.6)	1.49 (1.2, 1.9)	†
Professional, Scientific, Management	28.66 (25.5, 31.9)	2.50 (-2.0, 7.0)	1.15 (1.0, 1.4)	††	2.39 (1.6, 3.4)	0.12 (-1.3, 1.5)	1.72 (1.0, 2.9)	†	26.26 (23.2, 29.5)	2.39 (-2.0, 6.8)	1.15 (1.0, 1.4)	††
Educational Services, Health Care and Social Assistance	26.90 (24.9, 28.9)	1.90 (-1.1, 4.9)	1.12 (1.0, 1.3)	††	2.10 (1.3, 3.2)	-0.56 (-1.6, 0.5)	1.04 (0.7, 1.6)	†	24.80 (22.8, 26.9)	2.46 (-0.5, 5.4)	1.14 (1.0, 1.3)	††
Arts, Entertainment, Recreation, Accommodation, and Food Services	33.78 (28.2, 39.8)	5.15 (-2.9, 13.2)	1.37 (1.2, 1.6)	†	6.52 (4.2, 9.6)	-3.54 (-6.5, -0.5)	0.38 (0.2, 0.8)	††	27.26 (21.7, 33.4)	8.69 (0.5, 16.9)	1.44 (1.2, 1.8)	†
Other Services, Except Public Administration	30.33 (23.5, 37.9)	0.46 (-9.4, 10.3)	1.01 (0.7, 1.4)	††	3.17 (1.3, 6.3)	2.15 (-1.7, 6.0)	3.26 (1.5, 7.1)	†	27.17 (20.5, 34.7)	-1.68 (-11.7, 8.3)	0.89 (0.6, 1.3)	†††
Public Administration	20.90 (16.9, 25.4)	6.83 (1.3, 12.3)	0.82 (0.5, 1.3)	†††	1.21 (0.7, 2.0)	2.36 (0.1, 4.6)	3.02 (1.1, 8.4)	†	19.69 (15.7, 24.2)	4.47 (-1.0, 10.0)	0.70 (0.4, 1.1)	†††
**Main Occupation (2010 Census Codes)**												
Management, Business, and Financial	25.39 (22.6, 28.4)	0.72 (-4.6, 3.1)	1.11 (0.9, 1.4)	††	2.41 (1.7, 3.3)	-0.62 (-1.7,0.4)	0.77 (0.4, 1.6)	††	22.97 (20.4, 25.7)	-0.10 (-3.7, 3.5)	1.13 (0.9, 1.4)	††
Computer, Engineering, and Science	28.47 (24.9, 32.3)	3.65 (-1.0, 8.3)	1.11 (0.9, 1.3)	††	1.27 (0.6, 2.3)	0.85 (-0.5, 2.2)	4.00 (1.9, 8.3)	†	27.20 (23.7, 30.9)	2.80 (-1.7, 7.3)	1.08 (0.9, 1.3)	††
Education, Legal, Community Service, Arts, and Media	29.08 (25.8, 32.5)	-0.04 (-4.4, 4.4)	0.74 (0.6, 1.0)	†††	2.50 (1.3, 4.3)	-0.44 (-2.1, 1.2)	0.71 (0.3, 1.6)	††	26.57 (23.5, 29.8)	0.40 (-3.8, 4.6)	0.76 (0.6, 1.0)	†††
Healthcare Practitioners and Technical	21.98 (18.2, 26.1)	6.56 (0.9, 12.3)	1.70 (1.4, 2.1)	†	0.79 (0.3, 1.7)	0.33 (-0.7, 1.3)	1.68 (0.7, 4.3)	†	21.19 (17.3, 25.5)	6.23 (0.5, 12.0)	1.70 (1.4, 2.1)	†
Service	31.65 (28.1, 35.3)	1.64 (-3.6, 6.9)	1.23 (1.1, 1.4)	†	4.35 (2.8, 6.3)	-1.92 (-3.9, 0.0)	0.57 (0.3, 1.1)	††	27.30 (23.8, 31.0)	3.57 (-1.7, 8.8)	1.26 (1.1, 1.5)	†
Sales and Related	24.15 (20.0, 28.7)	11.10 (4.3, 17.8)	1.37 (1.0, 1.8)	††	1.83 (0.9, 3.2)	2.16 (-0.5, 4.9)	1.89 (0.9, 4.1)	†	22.32 (18.3, 26.8)	8.94 (2.3, 15.6)	1.33 (1.0, 1.8)	††
Office and Administrative Support	27.47 (23.8, 31.4)	3.95 (-1.0, 8.9)	1.08 (0.9, 1.3)	††	3.66 (2.6, 5.0)	-0.71 (-2.6, 1.2)	0.87 (0.4, 1.7)	††	23.81 (20.2, 27.8)	4.66 (-0.3, 9.6)	1.11 (0.9, 1.4)	††
Farming, Fishing, and Forestry	18.86 (9.6, 31.6)	2.53 (-12.0, 17.1)	0.75 (0.5, 1.2)	†	2.64 (0.3, 8.9)	-1.89 (-5.6, 1.9)	0.32 (0.1, 1.4)	†	16.23 (7.9, 28.2)	4.43 (-9.6, 18.4)	0.79 (0.5, 1.3)	†
Construction and Extraction	24.22 (15.6, 34.7)	1.59 (-10.4, 13.6)	1.26 (0.9, 1.8)	†	. .	-0.66 (-4.3, 3.0)	0.86 (0.2, 3.2)	†	22.28 (14.3, 32.0)	2.23 (-9.3, 13.7)	1.29 (0.9, 1.8)	†
Installation, Maintenance, and Repair	17.69 (9.8, 28.3)	2.57 (-8.7, 13.8)	1.19 (0.8, 1.8)	†	0.73 (0.2, 2.1)	1.95 (-1.0, 4.9)	3.09 (0.9, 10.5)	†	16.96 (9.1, 27.8)	0.62 (-10.6, 11.8)	1.09 (0.7, 1.7)	†
Production	19.70 (14.3, 26.1)	14.44 (5.3, 23.6)	1.46 (1.1, 1.9)	†	2.06 (0.1, 8.7)	-0.06 (-3.5, 3.3)	1.20 (0.6, 2.3)	†	17.64 (12.8, 23.4)	14.50 (5.7, 23.3)	1.48 (1.1, 1.9)	†
Transportation and Material Moving	32.85 (24.3, 42.3)	-5.28 (-15.5, 4.9)	0.78 (0.6, 1.1)	††	4.47 (2.0, 8.4)	0.34 (-3.4, 4.1)	0.70 (0.3, 1.6)	††	28.38 (20.2, 37.8)	-5.62 (-15.7, 4.4)	0.78 (0.5, 1.1)	††

Notes:

Sexual orientation was dichotomized as heterosexual vs. non-heterosexual by collapsing the “gay, lesbian, or homosexual”, “bisexual,” “not sexual / celibate / none,” and “other” categories into non-heterosexual.

*Difficulty concentrating, Dressing/Bathing, and Doing errands were not available 2013-2018.

NH refers to Non-Hispanic

† designates intercept

†† designates linear

††† designates quadratic

Table S4. Study Population, All Demographics.

	2020	
	Workers Weighted % (CI)	Nonworkers Weighted % (CI)
**Unweighted N**	12982	8967
**Weighted N (CI), millions**	19.7 (19, 20)	10 (10, 10)
**Age, years**		
18-29	22.5 (22, 23)	18.7 (17.6, 19.8)
30-44	33.9 (33.4, 34.5)	10.7 (9.7, 11.7)
45-64	36.8 (36.2, 37.5)	21.7 (20.6, 22.8)
65+	6.7 (6.3, 7.1)	48.9 (47.6, 50.2)
**Sex**		
Male	52.8 (52.2, 53.4)	41.8 (40.6, 43)
Female	47.2 (46.6, 47.8)	58.2 (57, 59.4)
**Race/Ethnicity**		
Hispanic/Latino	41.8 (41, 42.5)	34.3 (32.8, 35.8)
White, NH	35.6 (35, 36.1)	43.9 (42.7, 45.2)
Black, NH	5.2 (4.8, 5.6)	6.1 (5.3, 6.8)
Asian, NH	13.7 (13.2, 14.2)	12.5 (11.5, 13.6)
AI/AN, NH/PI, 2+, NH	3.8 (3.5, 4.1)	3.1 (2.5, 3.7)
**Highest Education**		
Less than High School	12.9 (12.1, 13.6)	19.7 (18.5, 20.8)
Grade 12/ High School Diploma	19.1 (18.3, 19.8)	27.3 (25.8, 28.7)
Some College or AA Degree	21.2 (20.3, 22)	23.5 (22.3, 24.6)
BA or BS Degree/Some Graduate School	28 (27, 29)	18.7 (17.6, 19.7)
Masters Degree or Higher	18.9 (18.1, 19.7)	10.9 (10.3, 11.6)
**Family Poverty Threshold Level**		
0-138%	15.2 (14.2, 16.1)	27.5 (25.9, 29)
139-249%	15.5 (14.4, 16.6)	18.9 (17.5, 20.3)
250-399%	17.6 (16.6, 18.5)	17.7 (16.2, 19.2)
400%+	51.8 (50.7, 52.8)	35.9 (34.4, 37.4)
**Annual household income, $**		
<20,000	9.4 (8.5, 10.2)	20.1 (18.7, 21.5)
20,000-39,999	13.7 (12.7, 14.8)	22.4 (20.8, 24)
40,000-74,999	21.7 (20.5, 22.9)	22.6 (21.4, 23.8)
75,000-99,999	12.5 (11.6, 13.4)	10.5 (9.5, 11.6)
100,000-129,999	13.5 (12.6, 14.4)	9.9 (8.9, 11)
130,000+	29.2 (28.3, 30)	14.5 (13.4, 15.5)
**Disability**		
Blind/Deaf or Has Severe Vision/Hearing Problem	3.4 (2.9, 4)	10.7 (9.7, 11.7)
Difficulty Concentrating (2019-2020 only)	7.4 (6.8, 8.1)	12.4 (11.2, 13.6)
Difficulty Dressing or Bathing (2019-2020 only)	0.6 (0.4, 0.9)	5.5 (4.6, 6.3)
Difficulty Doing Errands Alone (2019-2020 only)	2.6 (2.1, 3)	11.2 (10.2, 12.3)
Any of the above disabilities	11.6 (10.7, 12.5)	26.6 (25.1, 28.1)
**Sexual Orientation (did not ask adults >70 y/o in 2013-2014)**		
Heterosexual	90.3 (89.4, 91.1)	90.5 (89.4, 91.5)
Not heterosexual	9.7 (8.9, 10.6)	9.5 (8.5, 10.6)
**Gender**		
Cisgender	99.3 (99.1, 99.5)	99.5 (99.2, 99.8)
Transgender or gender non-conforming	0.7 (0.5, 0.9)	0.5 (0.2, 0.8)
Gender unavailable/not asked	. .	. .
**Employer Type at Main Job**		
Private	66 (64.7, 67.2)	. .
Government	18 (17, 19)	. .
Self-employed	13.6 (12.7, 14.4)	. .
Family business or farm	2.5 (2, 3)	. .
**Main Industry (2010 Census Codes)**		
Agriculture, Forestry, Fishing, Hunting and Mining	1.5 (1.1, 1.8)	. .
Construction	5 (4.4, 5.7)	. .
Manufacturing	8.4 (7.5, 9.2)	. .
Wholesale Trade	1.9 (1.6, 2.3)	. .
Retail Trade	8.4 (7.7, 9.2)	. .
Transportation, Warehousing, and Utilities	4.9 (4.3, 5.5)	. .
Information	2.5 (2.2, 2.8)	. .
Finance and Insurance, Real Estate, Rental and Leasing	5.5 (5, 6)	. .
Professional, Scientific, Management	16.5 (15.4-17.5)	. .
Educational Services, Health Care and Social Assistance	24.2 (23.3-25.2)	. .
Arts, Entertainment, Recreation, Accommodation, and Food Services	7.4 (6.6-8.2)	. .
Other Services, Except Public Administration	4.4 (3.8-5)	. .
Public Administration	6.3 (5.8, 6.9)	. .
**Main Occupation (2010 Census Codes)**		
Management, Business, and Financial	14.4 (13.5, 15.3)	. .
Computer, Engineering, and Science	10.6 (9.9, 11.3)	. .
Education, Legal, Community Service, Arts, and Media	13.3 (12.5, 14)	. .
Healthcare Practitioners and Technical	5.1 (4.6, 5.6)	. .
Service	16.6 (15.3, 17.8)	. .
Sales and Related	7.6 (6.9, 8.2)	. .
Office and Administrative Support	11.9 (11.1, 12.7)	. .
Farming, Fishing, and Forestry	0.9 (0.6, 1.2)	. .
Construction and Extraction	3.7 (3, 4.3)	. .
Installation, Maintenance, and Repair	2.5 (1.9, 3)	. .
Production	4.8 (4.1, 5.5)	. .
Transportation and Material Moving	5.5 (4.9, 6.1)	. .

Footnotes:

Among workers, 2.6% (2.2, 2.9) of open-ended occupation information could not be census occupation coded.

Among workers, 1.8% (1.5, 2.2) of open-ended industry information could not be census occupation coded.

NH refers to Non-Hispanic

Table S5. Study Population for Individual Years, 2013-2020.

	All years (2013-2020)			2013		2014		2015		2016		2017		2018		2019		2020	
	Nonworkers	Workers	All adults	Nonworkers	Workers	Nonworkers	Workers	Nonworkers	Workers	Nonworkers	Workers	Nonworkers	Workers	Nonworkers	Workers	Nonworkers	Workers	Nonworkers	Workers
	Weighted % (CI)	Weighted % (CI)	Weighted % (CI)	Weighted % (CI)	Weighted % (CI)	Weighted % (CI)	Weighted % (CI)	Weighted % (CI)	Weighted % (CI)	Weighted % (CI)	Weighted % (CI)	Weighted % (CI)	Weighted % (CI)	Weighted % (CI)	Weighted % (CI)	Weighted % (CI)	Weighted % (CI)	Weighted % (CI)	Weighted % (CI)
**Unweighted N**	81065	87703	168768	10687	10037	10616	8900	9938	11096	10489	10566	10171	10982	10294	10883	9903	12257	8967	12982
**Weighted N (CI), millions**	81.6 (81, 83)	152.1 (151, 153)	233.7 (234, 234)	10 (10, 10)	18 (18, 18)	10.2 (10, 11)	18.3 (18, 19)	9.7 (9, 10)	19.3 (19, 20)	10.4 (10, 11)	18.9 (18, 19)	10.2 (10, 11)	19.2 (19, 20)	10.5 (10, 11)	19.2 (19, 20)	10.3 (10, 11)	19.3 (19, 20)	10 (10, 10)	19.7 (19, 20)
**Age, years**																			
18-29	17.4 (16.8, 18)	24.8 (24.5,25.1)	22.2 (22.2,22.2)	20.7 (19.1,22.4)	24.8 (23.9, 25.7)	19.3 (17.5, 21.1)	24.9 (23.9, 25.9)	15.1 (13.1, 17)	25.6 (24.7, 26.5)	15.4 (13.8, 16.9)	25.4 (24.5, 26.2)	16.1 (14.4, 17.8)	25.7 (24.8, 26.5)	16.5 (14.7, 18.2)	25.5 (24.6, 26.4)	17.5 (16.1, 18.9)	24.2 (23.4, 24.9)	18.7 (17.6, 19.8)	22.5 (22, 23)
30-44	14 (13.2, 14.7)	33.2 (32.7, 33.6)	26.5 (26.3, 26.6)	15.3 (13.9, 16.8)	33.8 (32.9, 34.8)	16.6 (14.8, 18.4)	32.9 (31.8, 34)	15.6 (13.6, 17.6)	32.1 (30.9, 33.3)	15.7 (13.4, 18)	33.5 (32.1, 34.9)	13.1 (11.5, 14.6)	32.5 (31.5, 33.5)	12.5 (11.1, 13.9)	32.7 (31.7, 33.8)	12.2 (11, 13.4)	33.7 (33.1, 34.4)	10.7 (9.7, 11.7)	33.9 (33.4, 34.5)
45-64	26.4 (25.8, 26.9)	36.4 (36, 36.7)	32.9 (32.7, 33)	27.7 (26.3, 29.2)	36.3 (35.3, 37.3)	25.9 (24.4, 27.4)	37.1 (36, 38.1)	28.5 (26.7, 30.3)	36.7 (35.5, 37.9)	28.7 (26.6, 30.8)	35.6 (34.3, 37)	27.2 (25.5, 28.9)	36.5 (35.5, 37.4)	26.8 (25.7, 27.9)	36.3 (35.5, 37)	24.3 (23, 25.6)	35.7 (34.9, 36.4)	21.7 (20.6, 22.8)	36.8 (36.2, 37.5)
65+	42.3 (41.6, 43)	5.7 (5.4, 5.9)	18.5 (18.5, 18.5)	36.2 (35.0, 37.4)	5.1 (4.7, 5.5)	38.1 (36.7, 39.6)	5.1 (4.6, 5.7)	40.8 (39.1, 42.6)	5.6 (5, 6.1)	40.2 (38.5, 42)	5.5 (4.7, 6.4)	43.7 (42.1, 45.2)	5.3 (4.8, 5.9)	44.2 (42.7, 45.8)	5.5 (4.9, 6.2)	46.1 (45, 47.1)	6.4 (6, 6.8)	48.9 (47.6, 50.2)	6.7 (6.3, 7.1)
**Sex**																			
Male	39.3 (38.7, 39.9)	54 (53.7, 54.4)	48.9 (48.9, 48.9)	37.8 (36.3, 39.2)	54.9 (54.0, 55.7)	37.3 (35.5, 39)	55.2 (54.2, 56.2)	39.8 (37.6, 41.9)	53.5 (52.4, 54.6)	39.1 (36.8, 41.3)	54.3 (53, 55.6)	38.5 (36.7, 40.3)	54.3 (53.3, 55.3)	39.7 (37.9, 41.6)	53.8 (52.7, 55)	40.7 (39.3, 42)	53.6 (52.9, 54.3)	41.8 (40.6, 43)	52.8 (52.2, 53.4)
Female	60.7 (60.1, 61.3)	46 (45.6, 46.3)	51.1 (51.1, 51.1)	62.2 (60.8, 63.7)	45.1 (44.3, 46.0)	62.7 (61, 64.5)	44.8 (43.8, 45.8)	60.2 (58.1, 62.4)	46.5 (45.4, 47.6)	60.9 (58.7, 63.2)	45.7 (44.4, 47)	61.5 (59.7, 63.3)	45.7 (44.7, 46.7)	60.3 (58.4, 62.1)	46.2 (45, 47.3)	59.3 (58, 60.7)	46.4 (45.7, 47.1)	58.2 (57, 59.4)	47.2 (46.6, 47.8)
**Race/Ethnicity**																			
Hispanic/Latino	32.1 (31.3, 32.9)	38.6 (38.2, 39)	36.3 (36.3, 36.3)	31.4 (29.6, 33.3)	36.5 (35.5, 37.5)	32.3 (30.4, 34.1)	36.4 (35.4, 37.4)	31.1 (29.2, 33.1)	37.2 (36.2, 38.2)	31.6 (29.4, 33.8)	37.7 (36.5, 38.9)	31.9 (29.9, 33.9)	37.8 (36.8, 38.9)	30.5 (28.3, 32.7)	39.2 (38, 40.3)	33.6 (32, 35.3)	42.1 (41.2, 42.9)	34.3 (32.8, 35.8)	41.8 (41, 42.5)
White, NH	45.4 (44.6, 46.2)	38.7 (38.3, 39.1)	41 (41, 41)	44.9 (43.1, 46.7)	42.0 (40.9, 43.0)	45.3 (43.1, 47.6)	41 (39.8, 42.2)	47.3 (45.2, 49.4)	39.5 (38.5, 40.6)	45.6 (43, 48.2)	39.5 (38.1, 40.9)	47 (44.9, 49.1)	38.3 (37.2, 39.3)	44.7 (43, 46.5)	38.8 (37.9, 39.7)	44.1 (42.7, 45.4)	35.6 (34.9, 36.2)	43.9 (42.7, 45.2)	35.6 (35, 36.1)
Black, NH	6.1 (5.7, 6.4)	5.3 (5.1, 5.5)	5.6 (5.6, 5.6)	6.8 (5.8, 7.8)	5.0 (4.4, 5.5)	5.7 (4.7, 6.6)	5.6 (5.1, 6.1)	6.1 (5.2, 7.1)	5.4 (4.9, 5.9)	6 (5.1, 6.9)	5.4 (4.9, 5.9)	5.7 (4.7, 6.8)	5.5 (4.9, 6)	5.7 (4.8, 6.6)	5.4 (5, 5.9)	6.4 (5.7, 7.1)	5 (4.6, 5.4)	6.1 (5.3, 6.8)	5.2 (4.8, 5.6)
Asian, NH	13.5 (12.9, 14)	14.3 (14, 14.6)	14 (14, 14)	13.6 (12.2, 14.9)	14.0 (13.2, 14.8)	13.7 (12, 15.3)	14.3 (13.4, 15.2)	12.6 (11.2, 14)	15 (14.3, 15.7)	13.8 (11.9, 15.8)	14.5 (13.4, 15.6)	12.4 (10.9, 13.9)	15.6 (14.8, 16.3)	16.6 (14.9, 18.2)	13.4 (12.5, 14.3)	12.4 (11.5, 13.3)	13.9 (13.4, 14.4)	12.5 (11.5, 13.6)	13.7 (13.2, 14.2)
AI/AN, NH/PI, 2+, NH	3 (2.8, 3.2)	3.1 (3, 3.2)	3.1 (3, 3.1)	3.3 (2.8, 3.8)	2.6 (2.3, 2.9)	3.0 (2.3, 3.8)	2.8 (2.3, 3.2)	2.8 (2.3, 3.4)	2.9 (2.6, 3.2)	3.0 (2.3, 3.7)	2.9 (2.5, 3.3)	2.9 (2.2, 3.6)	2.9 (2.5, 3.2)	2.5 (1.9, 3.1)	3.2 (2.9, 3.5)	3.5 (3, 4)	3.5 (3.2, 3.8)	3.1 (2.5, 3.7)	3.8 (3.5, 4.1)
**Highest Education**																			
Less than High School	20.9 (20.3, 21.5)	13.3 (12.9, 13.6)	15.9 (15.8, 16)	19.5 (18.0, 21.0)	13.2 (12.3, 14.0)	19.4 (17.6, 21.1)	12.8 (11.8, 13.7)	22 (20.1, 24)	14.8 (13.8, 15.9)	21.9 (19.9, 23.8)	14.4 (13.3, 15.4)	21.4 (19.8, 23)	13.4 (12.5, 14.4)	23.3 (21.6, 24.9)	12.7 (11.8, 13.6)	19.9 (18.3, 21.4)	11.9 (11.1, 12.7)	19.7 (18.5, 20.8)	12.9 (12.1, 13.6)
Grade 12/ High School Diploma	26.6 (26, 27.2)	20.3 (20, 20.6)	22.5 (22.4, 22.6)	29.2 (27.7, 30.7)	21.7 (20.8, 22.6)	27.5 (25.9, 29.1)	22.5 (21.6, 23.4)	25.2 (24, 26.4)	20.3 (19.7, 20.9)	26.2 (24.1, 28.4)	19.7 (18.5, 20.8)	25.7 (24.1, 27.3)	19.8 (19, 20.7)	25.5 (23.7, 27.3)	19.6 (18.6, 20.6)	26.4 (25, 27.9)	20.1 (19.3, 20.9)	27.3 (25.8, 28.7)	19.1 (18.3, 19.8)
Some College or AA Degree	24.1 (23.5, 24.8)	23.3 (22.8, 23.9)	23.6 (23.2, 24)	25.9 (24.0, 27.8)	26.1 (24.8, 27.3)	27.6 (25.5, 29.8)	24.8 (23.4, 26.2)	24.7 (22.6, 26.9)	23.6 (22, 25.1)	22.7 (20.5, 24.8)	23.6 (21.7, 25.5)	23 (20.9, 25.1)	22 (20.6, 23.3)	22 (20.3, 23.8)	23.5 (21.9, 25.2)	23.7 (22.5, 24.9)	22.4 (21.7, 23.2)	23.5 (22.3, 24.6)	21.2 (20.3, 22)
BA or BS Degree/Some Graduate School	18.1 (17.5, 18.7)	26.8 (26.3, 27.3)	23.8 (23.4, 24.1)	16.6 (15.1, 18.2)	24.5 (23.0, 25.9)	16.8 (15.1, 18.5)	25.7 (24.4, 27)	18 (16.4, 19.6)	26.7 (25.1, 28.4)	19.2 (16.7, 21.7)	27.2 (25.4, 29)	18.9 (17, 20.9)	28.3 (26.9, 29.7)	18.1 (16.2, 19.9)	27.5 (26, 28.9)	18.8 (17.8, 19.7)	26.3 (25.3, 27.2)	18.7 (17.6, 19.7)	28 (27, 29)
Masters Degree or Higher	10.2 (9.8, 10.6)	16.3 (15.9, 16.7)	14.2 (13.9, 14.5)	8.8 (7.9, 9.6)	14.6 (13.6, 15.6)	8.7 (7.6, 9.9)	14.2 (13.1, 15.3)	10.1 (8.7, 11.5)	14.6 (13.2, 15.9)	10.1 (8.5, 11.6)	15.2 (13.4, 16.9)	10.9 (9.7, 12.2)	16.5 (15.3, 17.7)	11.2 (10.1, 12.3)	16.8 (15.5, 18)	11.2 (10.5, 12)	19.3 (18.5, 20.2)	10.9 (10.3, 11.6)	18.9 (18.1, 19.7)
**Family Poverty Threshold Level**																			
0-138%	32.7 (31.7, 33.6)	19.3 (18.8, 19.9)	24 (23.5, 24.5)	35.7 (33.9, 37.5)	18.8 (17.3, 20.3)	34.9 (32.1, 37.7)	21.5 (19.7, 23.2)	36.1 (34, 38.1)	22.5 (20.7, 24.2)	35.0 (32, 38)	21.6 (19.6, 23.7)	29.4 (27.5, 31.2)	20 (18.5, 21.4)	33.2 (30.6, 35.7)	18.8 (17.3, 20.3)	29.7 (28, 31.5)	16.6 (15.5, 17.7)	27.5 (25.9, 29)	15.2 (14.2, 16.1)
139-249%	18.8 (18.2, 19.4)	16.6 (16, 17.1)	17.3 (16.9, 17.8)	17.4 (15.9, 18.9)	17.6 (16.1, 19.1)	20.4 (18.5, 22.3)	17.4 (15.7, 19.1)	18 (16.3, 19.6)	17.3 (15.9, 18.7)	18.1 (16, 20.3)	16.8 (15.1, 18.6)	18.9 (16.9, 20.9)	16.3 (14.6, 18.1)	19 (17.1, 21)	16.4 (14.8, 17.9)	19.6 (18.2, 21)	15.4 (14.3, 16.5)	18.9 (17.5, 20.3)	15.5 (14.4, 16.6)
250-399%	16.5 (15.8, 17.2)	17.5 (16.9, 18)	17.1 (16.7, 17.5)	18.3 (16.8, 19.7)	18.6 (17.3, 19.8)	17.4 (15.7, 19.2)	17.6 (16, 19.2)	15.4 (13.5, 17.3)	16.8 (15.2, 18.4)	14 (11.8, 16.2)	17.5 (15.4, 19.5)	16.6 (14.5, 18.8)	15.2 (14.1, 16.4)	14.6 (12.5, 16.7)	18.0 (16.1, 19.8)	18 (16.7, 19.3)	18.6 (17.6, 19.5)	17.7 (16.2, 19.2)	17.6 (16.6, 18.5)
400%+	32 (31.3, 32.8)	46.6 (46, 47.2)	41.5 (41.1, 42)	28.6 (26.7, 30.5)	45.0 (43.7, 46.4)	27.3 (25.3, 29.2)	43.5 (41.6, 45.5)	30.6 (28.7, 32.5)	43.4 (41.8, 45.1)	32.9 (29.8, 36)	44.1 (42.0, 46.2)	35.1 (32.7, 37.4)	48.4 (46.7, 50.2)	33.2 (31.1, 35.2)	46.9 (45.2, 48.6)	32.7 (31, 34.3)	49.4 (48.1, 50.8)	35.9 (34.4, 37.4)	51.8 (50.7, 52.8)
**Annual household income, $**																			
<20,000	24.7 (23.8, 25.6)	12 (11.5, 12.4)	16.4 (16, 16.9)	28.2 (26.3, 30.1)	11.0 (9.7, 12.2)	27 (24.6, 29.4)	13.8 (12.4, 15.1)	28.9 (27.1, 30.7)	15.9 (14.4, 17.4)	26.9 (24.6, 29.2)	14.0 (12.0, 16.0)	21.4 (19.4, 23.4)	11.5 (10.5, 12.6)	23.7 (21.6, 25.8)	10.0 (9.0, 11.1)	21.4 (20, 22.8)	10.3 (9.4, 11.2)	20.1 (18.7, 21.5)	9.4 (8.5, 10.2)
20,000-39,999	23 (22.3, 23.8)	16.8 (16.3, 17.3)	19 (18.6, 19.4)	24.6 (22.8, 26.4)	19.9 (18.3, 21.5)	25.4 (23.6, 27.3)	19.3 (17.9, 20.7)	22.9 (21.2, 24.5)	18 (16.6, 19.3)	23.2 (20.8, 25.7)	18.2 (16.2, 20.2)	20.9 (18.6, 23.2)	16.2 (15, 17.4)	21.7 (19.4, 24.1)	15.9 (14.6, 17.1)	23.1 (21.4, 24.8)	13.6 (12.5, 14.7)	22.4 (20.8, 24)	13.7 (12.7, 14.8)
40,000-74,999	22.6 (21.8, 23.3)	22.8 (22.3, 23.4)	22.7 (22.3, 23.2)	22.1 (20.7, 23.5)	24.2 (22.7, 25.7)	23 (20.8, 25.2)	22.6 (20.9, 24.2)	21.5 (19.4, 23.6)	23.1 (21.3, 24.9)	21.2 (19, 23.4)	24.0 (21.7, 26.3)	23.6 (21.5, 25.7)	21.3 (19.9, 22.6)	22.7 (20.4, 25)	22.7 (21.2, 24.1)	23.7 (22.3, 25.2)	23.2 (22.1, 24.4)	22.6 (21.4, 23.8)	21.7 (20.5, 22.9)
75,000-99,999	9.3 (8.8, 9.7)	11.5 (11.1, 12)	10.7 (10.4, 11.1)	7.8 (6.9, 8.8)	12.0 (10.8, 13.1)	7.5 (6.4, 8.6)	11 (9.8, 12.2)	8.8 (7.4, 10.1)	10.6 (9.2, 12.1)	8.6 (7.2, 9.9)	11.5 (9.9, 13.1)	11.2 (9.6, 12.7)	11.2 (10.1, 12.4)	9 (7.6, 10.4)	10.9 (9.6, 12.1)	10.8 (9.5, 12.1)	12.6 (11.6, 13.5)	10.5 (9.5, 11.6)	12.5 (11.6, 13.4)
100,000-129,999	8.9 (8.4, 9.4)	12.1 (11.7, 12.5)	11 (10.6, 11.3)	7.9 (6.8, 9.1)	12.0 (11.1, 13.0)	7.8 (6.6, 8.9)	11.5 (10.4, 12.7)	8.7 (7.2, 10.3)	12 (10.7, 13.2)	8.9 (7, 10.7)	10.3 (9.0, 11.6)	9.2 (7.9, 10.4)	12.3 (11.1, 13.4)	9.6 (8.3, 10.9)	12.0 (10.5, 13.4)	9.3 (8.4, 10.1)	13 (12.1, 13.8)	9.9 (8.9, 11)	13.5 (12.6, 14.4)
130,000+	11.6 (11, 12.1)	24.8 (24.3, 25.3)	20.2 (19.8, 20.5)	9.4 (8.2, 10.6)	21.0 (19.9, 22.1)	9.3 (7.8, 10.7)	21.9 (20.2, 23.5)	9.3 (7.8, 10.7)	20.4 (19.2, 21.6)	11.2 (9.2, 13.3)	22.1 (20.1, 24.1)	13.7 (12, 15.4)	27.5 (25.9, 29.1)	13.2 (11.7, 14.8)	28.6 (27, 30.2)	11.7 (10.9, 12.5)	27.4 (26.3, 28.4)	14.5 (13.4, 15.5)	29.2 (28.3, 30)
**Disability**																			
Blind/Deaf or Has Severe Vision/Hearing Problem																			
Yes	12.1 (11.5, 12.6)	4.4 (4.1, 4.7)	7.1 (6.8, 7.3)	11.7 (10.6, 12.8)	4.3 (3.7, 4.9)	11 (9.7, 12.3)	4.4 (3.6, 5.1)	14.5 (12.9, 16.1)	5.6 (4.5, 6.8)	13.1 (10.9, 15.2)	4.3 (3.5, 5.1)	13.1 (11.6, 14.5)	5.3 (4.4, 6.2)	12 (10.5, 13.5)	4.7 (3.9, 5.4)	10.5 (9.2, 11.7)	3.1 (2.6, 3.7)	10.7 (9.7, 11.7)	3.4 (2.9, 4)
No	87.9 (87.4, 88.5)	95.6 (95.3, 95.9)	92.9 (92.7, 93.2)	88.2 (87.1, 89.3)	95.7 (95.1, 96.3)	89 (87.7, 90.3)	95.6 (94.9, 96.4)	85.5 (83.9, 87.1)	94.4 (93.2, 95.5)	86.9 (84.8, 89.1)	95.7 (94.9, 96.5)	86.9 (85.5, 88.4)	94.7 (93.8, 95.6)	88 (86.5, 89.5)	95.3 (94.6, 96.1)	89.5 (88.3, 90.8)	96.9 (96.3, 97.4)	89.3 (88.3, 90.3)	96.6 (96, 97.1)
Difficulty Concentrating?																			
Yes	3.3 (3.1, 3.5)	1.7 (1.6, 1.9)	2.3 (2.2, 2.4)	. .	. .	. .	. .	. .	. .	. .	. .	. .	. .	. .	. .	14.1 (12.9, 15.3)	6.1 (5.5, 6.8)	12.4 (11.2, 13.6)	7.4 (6.8, 8.1)
No	21.6 (21.2, 22)	23.9 (23.7, 24.1)	23.1 (23, 23.2)	. .	. .	. .	. .	. .	. .	. .	. .	. .	. .	. .	. .	85.9 (84.7, 87.1)	93.9 (93.2, 94.5)	87.6 (86.4, 88.8)	92.6 (91.9, 93.2)
Difficulty Dressing or Bathing																			
Yes	1.5 (1.3, 1.6)	0.2 (0.1, 0.2)	0.6 (0.6, 0.7)	. .	. .	. .	. .	. .	. .	. .	. .	. .	. .	. .	. .	6.2 (5.4, 7)	0.8 (0.4, 1.2)	5.5 (4.6, 6.3)	0.6 (0.4, 0.9)
No	23.5 (23.1, 23.9)	25.5 (25.2, 25.7)	24.8 (24.7, 24.8)	. .	. .	. .	. .	. .	. .	. .	. .	. .	. .	. .	. .	93.8 (93, 94.6)	99.2 (98.8, 99.6)	94.5 (93.7, 95.4)	99.4 (99.1, 99.6)
Difficulty Doing Errands Alone																			
Yes	2.9 (2.7, 3.1)	0.7 (0.6, 0.8)	1.5 (1.4, 1.5)	. .	. .	. .	. .	. .	. .	. .	. .	. .	. .	. .	. .	12.1 (11, 13.3)	2.7 (2.2, 3.3)	11.2 (10.2, 12.3)	2.6 (2.1, 3)
No	22 (21.6, 22.4)	25 (24.7, 25.2)	23.9 (23.9, 24)	. .	. .	. .	. .	. .	. .	. .	. .	. .	. .	. .	. .	87.9 (86.7, 89)	97.3 (96.7, 97.8)	88.8 (87.7, 89.8)	97.4 (97, 97.9)
Any of the above disabilities	16.1 (15.5, 16.7)	6.3 (6, 6.7)	9.8 (9.5, 10)	11.7 (10.6, 12.8)	4.3 (3.7, 4.9)	11 (9.7, 12.3)	4.4 (3.6, 5.1)	14.5 (12.9, 16.1)	5.6 (4.5, 6.8)	13.1 (10.9, 15.2)	4.3 (3.5, 5.1)	13.1 (11.6, 14.5)	5.3 (4.4, 6.2)	12 (10.5, 13.5)	4.7 (3.9, 5.4)	27.2 (25.7, 28.6)	10.2 (9.2, 11.2)	26.6 (25.1, 28.1)	11.6 (10.7, 12.5)
**Sexual Orientation**																			
Heterosexual	92.8 (92.4, 93.3)	92.5 (92.2, 92.9)	92.6 (92.4, 92.9)	95.2 (94.2, 96.2)	94.9 (94.0, 95.7)	95 (93.7, 96.4)	94.8 (93.7, 95.8)	91.3 (89.8, 92.9)	93.3 (92.1, 94.4)	94 (92.5, 95.6)	93.0 (91.6, 94.3)	93.5 (92.2, 94.8)	93.5 (92.7, 94.2)	94.2 (92.9, 95.4)	91.7 (90.6, 92.8)	89.8 (88.7, 90.9)	89.4 (88.6, 90.3)	90.5 (89.4, 91.5)	90.3 (89.4, 91.1)
Not heterosexual	7.2 (6.7, 7.6)	7.5 (7.1, 7.8)	7.4 (7.1, 7.6)	4.7 (3.7, 5.7)	5.1 (4.3, 6.0)	5 (3.6, 6.3)	5.2 (4.2, 6.3)	8.7 (7.1, 10.2)	6.7 (5.6, 7.9)	6 (4.4, 7.5)	7.0 (5.7, 8.4)	6.5 (5.2, 7.8)	6.5 (5.8, 7.3)	5.8 (4.6, 7.1)	8.3 (7.2, 9.4)	10.2 (9.1, 11.3)	10.6 (9.7, 11.4)	9.5 (8.5, 10.6)	9.7 (8.9, 10.6)
**Gender**																			
Cisgender	50 (49.3, 50.6)	50.6 (50.3, 51)	50.4 (50.3, 50.5)	. .	. .	. .	. .	. .	. .	. .	. .	99.5 (99, 99.9)	99.6 (99.3, 99.8)	99.7 (99.3, 100)	99.7 (99.5, 99.9)	98.6 (98.1, 99.1)	99.3 (99, 99.6)	99.5 (99.2, 99.8)	99.3 (99.1, 99.5)
Transgender or gender non-conforming	0.3 (0.2, 0.5)	0.3 (0.2, 0.4)	0.3 (0.2, 0.4)	. .	. .	. .	. .	. .	. .	. .	. .	0.5 (0.1, 1)	0.4 (0.2, 0.7)	0.3 (0, 0.7)	0.3 (0.1, 0.5)	1.4 (0.9, 1.9)	0.7 (0.4, 1)	0.5 (0.2, 0.8)	0.7 (0.5, 0.9)
Gender unavailable/not asked	49.7 (49.1, 50.3)	49.1 (48.8, 49.4)	49.3 (49.3, 49.3)	. .	. .	. .	. .	. .	. .	. .	. .	. .	. .	. .	. .	. .	. .	. .	. .

Notes: Sexual Orientation not asked of adults >70 y/o in 2013-2014; Difficulty concentrating, Dressing/Bathing, and Doing errands were not available 2013-2018; NH refers to Non-Hispanic

Table S6. Study Population for Individual Years, Employment Information 2013-2020.

	All years (2013-2020)		2013	2014	2015	2016	2017	2018	2019	2020
	Workers Weighted % (CI)	All adults Weighted % (CI)	Workers Weighted % (CI)	Workers Weighted % (CI)	Workers Weighted % (CI)	Workers Weighted % (CI)	Workers Weighted % (CI)	Workers Weighted % (CI)	Workers Weighted % (CI)	Workers Weighted % (CI)
**Unweighted N**	87703	168768	10037	8900	11096	10566	10982	10883	12257	12982
**Weighted N (CI), millions**	152.1 (151, 153)	233.7 (234, 234)	18 (18, 18)	18.3 (18, 19)	19.3 (19, 20)	18.9 (18, 19)	19.2 (19, 20)	19.2 (19, 20)	19.3 (19, 20)	19.7 (19, 20)
**Employer Type at Main Job**										
Private	65.9 (65.3, 66.5)	65.9 (65.3, 66.5)	65.5 (64.0, 67.1)	66.5 (64.7, 68.4)	66.5 (64.2, 68.8)	65.6 (63.2, 68)	65.4 (63.6, 67.2)	65.3 (63.5, 67.2)	66.4 (65, 67.8)	66 (64.7, 67.2)
Government	16.1 (15.7, 16.6)	16.1 (15.7, 16.6)	15.8 (14.5, 17.0)	15.2 (13.9, 16.5)	15.1 (13.7, 16.4)	15 (13.2, 16.7)	16.3 (15.1, 17.5)	16.5 (15.4, 17.6)	17.1 (16.2, 18.1)	18 (17, 19)
Self-employed	15.7 (15.1, 16.2)	15.7 (15.1, 16.2)	16.9 (15.6, 18.2)	16.9 (15.4, 18.5)	15.9 (14.3, 17.4)	17.3 (15, 19.5)	16.2 (14.7, 17.7)	15.4 (13.9, 16.9)	13.3 (12.5, 14.1)	13.6 (12.7, 14.4)
Family business or farm	2.3 (2.1, 2.5)	2.3 (2.1, 2.5)	1.8 (1.4, 2.2)	1.4 (0.9, 1.8)	2.5 (2, 3.1)	2.2 (1.4, 2.9)	2.1 (1.6, 2.6)	2.7 (2, 3.4)	3.2 (2.7, 3.7)	2.5 (2, 3)
**Main Industry (2010 Census Codes)**										
Agriculture, Forestry, Fishing, Hunting and Mining	2.4 (2.2, 2.6)	2.4 (2.2, 2.6)	2.6 (2.2, 3.1)	2.7 (2.1, 3.3)	2.8 (2.3, 3.2)	2.6 (2.1, 3.2)	2.8 (2.3, 3.3)	2.5 (1.8, 3.1)	1.5 (1.3, 1.8)	1.5 (1.1, 1.8)
Construction	6.3 (6, 6.7)	6.3 (6, 6.7)	6.8 (5.9, 7.7)	5.8 (4.7, 6.9)	6.3 (5.4, 7.3)	7.3 (6.1, 8.6)	7.1 (6.2, 8)	6.8 (5.8, 7.9)	5.2 (4.5, 5.8)	5 (4.4, 5.7)
Manufacturing	9.3 (8.9, 9.8)	9.3 (8.9, 9.8)	11.9 (10.6, 13.2)	10.7 (9.2, 12.1)	10.4 (9.2, 11.7)	10 (8.2, 11.8)	7.3 (6.3, 8.3)	7.7 (6.7, 8.7)	8.5 (7.6, 9.3)	8.4 (7.5, 9.2)
Wholesale Trade	2 (1.7, 2.2)	1.9 (1.7, 2.2)	2.2 (1.6, 2.8)	2.2 (1.7, 2.8)	2.1 (1.6, 2.6)	1.6 (1, 2.2)	2.2 (1.6, 2.8)	1.7 (1.1, 2.2)	1.6 (1.3, 2)	1.9 (1.6, 2.3)
Retail Trade	9.3 (8.9, 9.8)	9.3 (8.9, 9.8)	9.5 (8.5, 10.4)	11.4 (10.1, 12.7)	9.8 (8.6, 10.9)	9.9 (8, 11.8)	8.3 (7.3, 9.3)	9 (7.8, 10.3)	8.4 (7.5, 9.3)	8.4 (7.7, 9.2)
Transportation, Warehousing, and Utilities	4.5 (4.2, 4.8)	4.5 (4.2, 4.8)	4.1 (3.4, 4.9)	5 (4.1, 6)	4.6 (3.6, 5.6)	4.1 (3.2, 5.1)	4.6 (3.8, 5.4)	4.4 (3.5, 5.3)	4.4 (3.7, 5.1)	4.9 (4.3, 5.5)
Information	2.5 (2.3, 2.7)	2.5 (2.3, 2.7)	2.9 (2.4, 3.5)	2.6 (1.8, 3.3)	2.5 (1.9, 3)	2.7 (1.8, 3.7)	2.6 (2, 3.2)	2 (1.4, 2.6)	2.3 (1.9, 2.6)	2.5 (2.2, 2.8)
Finance and Insurance, Real Estate, Rental and Leasing	6 (5.6, 6.3)	6 (5.6, 6.3)	6.6 (5.7, 7.4)	7.2 (6.1, 8.3)	6.2 (5.1, 7.2)	5.7 (4.4, 6.9)	5.5 (4.7, 6.3)	5.7 (4.7, 6.6)	5.5 (4.9, 6)	5.5 (5, 6)
Professional, Scientific, Management	14.6 (14.1, 15)	14.6 (14.1, 15)	13.6 (12.2, 14.9)	12.3 (10.9, 13.8)	13.7 (12.4, 15)	13.6 (12.1, 15)	15.3 (14.1, 16.6)	15.2 (14, 16.4)	16.5 (15.5, 17.5)	16.5 (15.4, 17.5)
Educational Services, Health Care and Social Assistance	22.9 (22.4, 23.4)	22.9 (22.4, 23.4)	21.1 (19.8, 22.5)	21.1 (19.7, 22.5)	21.1 (19.5, 22.8)	22.8 (21, 24.6)	23.3 (21.7, 25)	23.8 (22.3, 25.3)	25.9 (24.7, 27)	24.2 (23.3, 25.2)
Arts, Entertainment, Recreation, Accommodation, and Food Services	7.9 (7.5, 8.3)	7.9 (7.5, 8.3)	7.2 (6.4, 8.0)	6.6 (5.5, 7.6)	8.4 (7.1, 9.6)	8.7 (6.9, 10.5)	9 (7.8, 10.2)	8.4 (7.4, 9.4)	7.4 (6.5, 8.2)	7.4 (6.6, 8.2)
Other Services, Except Public Administration	4.9 (4.6, 5.2)	4.9 (4.6, 5.2)	4.7 (3.8, 5.5)	5.3 (4.4, 6.3)	5 (4, 6.1)	4.8 (3.7, 5.9)	5.4 (4.5, 6.4)	5.3 (4.4, 6.1)	4.4 (3.8, 5.1)	4.4 (3.8, 5)
Public Administration	5.4 (5.1, 5.7)	5.4 (5.1, 5.7)	5.4 (4.7, 6.1)	5.3 (4.5, 6.1)	5.5 (4.4, 6.7)	4.7 (3.6, 5.7)	4.9 (4.3, 5.6)	5.1 (4.3, 5.8)	5.9 (5.4, 6.5)	6.3 (5.8, 6.9)
Could not be industry coded	1.3 (1.1, 1.4)	1.3 (1.1, 1.4)	1.2 (0.8, 1.6)	1.4 (0.9, 1.9)	0.6 (0.3, 0.9)	0.4 (0.1, 0.6)	1.1 (0.7, 1.5)	2.0 (1.4, 2.5)	1.6 (1.2, 2)	1.8 (1.5, 2.2)
**Main Occupation (2010 Census Codes)**										
Management, Business, and Financial	13.4 (13, 13.9)	13.4 (13, 13.9)	14.2 (13.1, 15.3)	14.8 (13.4, 16.3)	13.0 (11.4, 14.6)	12 (10.3, 13.8)	13.1 (11.9, 14.3)	12.5 (11.3, 13.8)	13.6 (12.9, 14.3)	14.4 (13.5, 15.3)
Computer, Engineering, and Science	8.8 (8.4, 9.1)	8.8 (8.4, 9.1)	7.3 (6.3, 8.3)	7.8 (6.6, 9)	7.5 (6.5, 8.5)	7.6 (6.4, 8.8)	9.7 (8.8, 10.6)	8.4 (7.5, 9.4)	11.4 (10.6, 12.2)	10.6 (9.9, 11.3)
Education, Legal, Community Service, Arts, and Media	13.4 (12.9, 13.9)	13.4 (12.9, 13.9)	13.3 (12.2, 14.4)	12.5 (11.2, 13.8)	14.0 (12.6, 15.4)	13.3 (11.8, 14.8)	13.9 (12.5, 15.2)	13.7 (12.3, 15.2)	13 (12.2, 13.8)	13.3 (12.5, 14)
Healthcare Practitioners and Technical	5.0 (4.7, 5.3)	5.0 (4.7, 5.3)	5.1 (4.3, 5.8)	4.4 (3.7, 5)	5.0 (4.1, 5.9)	4.9 (3.7, 6)	4.7 (3.7, 5.6)	5 (4.1, 5.9)	6.1 (5.5, 6.7)	5.1 (4.6, 5.6)
Service	17.3 (16.7, 17.8)	17.3 (16.7, 17.8)	17.0 (15.5, 18.4)	18.1 (16.6, 19.6)	17.5 (16.2, 18.8)	17.7 (15.6, 19.9)	17.1 (15.4, 18.9)	17.7 (16, 19.5)	16.5 (15.3, 17.7)	16.6 (15.3, 17.8)
Sales and Related	8.5 (8.1, 8.9)	8.5 (8.1, 8.9)	8.8 (7.8, 9.8)	9.6 (8.2, 10.9)	8.5 (7.4, 9.7)	9.2 (7.6, 10.8)	8.3 (7.1, 9.5)	8.7 (7.6, 9.8)	7.3 (6.5, 8.1)	7.6 (6.9, 8.2)
Office and Administrative Support	11.4 (10.9, 11.9)	11.4 (10.9, 11.9)	12.9 (11.8, 14.1)	11 (9.7, 12.3)	10.6 (9.5, 11.7)	11.3 (9.4, 13.2)	10.7 (9.4, 12)	10.4 (9.1, 11.7)	12.5 (11.6, 13.3)	11.9 (11.1, 12.7)
Farming, Fishing, and Forestry	1.4 (1.3, 1.6)	1.4 (1.3, 1.6)	1.6 (1.2, 2.0)	1.7 (1.2, 2.2)	1.4 (1.1, 1.8)	1.7 (1.3, 2.1)	1.9 (1.4, 2.3)	1.5 (1, 2)	0.8 (0.6, 1.1)	0.9 (0.6, 1.2)
Construction and Extraction	4.9 (4.6, 5.3)	5 (4.6, 5.3)	4.8 (4.0, 5.6)	4.5 (3.5, 5.5)	5.5 (4.6, 6.4)	6 (4.9, 7.1)	5.5 (4.7, 6.4)	5.6 (4.6, 6.6)	3.8 (3.1, 4.5)	3.7 (3, 4.3)
Installation, Maintenance, and Repair	2.5 (2.3, 2.7)	2.5 (2.3, 2.7)	2.6 (2.0, 3.2)	2.3 (1.8, 2.8)	2.5 (1.9, 3.1)	2.3 (1.5, 3.2)	2.7 (2.2, 3.1)	2.4 (1.9, 2.9)	2.8 (2.2, 3.4)	2.5 (1.9, 3)
Production	5.6 (5.3, 5.9)	5.6 (5.3, 5.9)	6.6 (5.6, 7.7)	6.8 (5.8, 7.8)	5.6 (4.6, 6.5)	6 (4.6, 7.5)	5.5 (4.7, 6.4)	5.2 (4.4, 6)	4.4 (3.8, 5)	4.8 (4.1, 5.5)
Transportation and Material Moving	5.9 (5.6, 6.3)	5.9 (5.6, 6.3)	4.8 (4.0, 5.5)	5.5 (4.6, 6.4)	7.9 (6.7, 9.1)	7.1 (5.7, 8.5)	5.4 (4.7, 6.1)	5.1 (4.3, 6)	5.9 (5.0, 6.8)	5.5 (4.9, 6.1)
Could not be occupation coded	1.5 (1.3, 1.7)	1.5 (1.4, 1.7)	0.9 (0.6, 1.2)	1.0 (0.5, 1.4)	0.8 (0.4, 1.2)	0.6 (0.3, 1)	1.3 (0.9, 1.7)	3.2 (2.5, 4)	1.6 (1.3, 1.9)	2.6 (2.2, 2.9)

Table S7. Suicidal Ideation in Working Adults.

	Suicidal Ideation in the Past Year Among All Workers				Suicidal Ideation in the Past Year Among Workers Who Have Ever Thought of Suicide			
	Prevalence in 2019, % (95% CI)	Absolute Change in Prevalence from 2019 to 2020, % (95% CI)	Trend Adjusted Prevalence Ratio, PR (CI)	Model Type	Prevalence in 2019, % (95% CI)	Absolute Change in Prevalence from 2019 to 2020, % (95% CI)	Trend Adjusted Prevalence Ratio, PR (CI)	Model Type
**All Workers**	4.43 (3.9, 5.1)	-0.20 (-1.0, 0.6)	0.75 (0.6, 0.9)	††	31.41 (27.7, 35.3)	1.71 (-3.5, 7.0)	0.98 (0.8, 1.2)	††

**Age**								
18-29	8.84 (7.0, 11.0)	1.31 (-1.4, 4.0)	0.82 (0.6, 1.1)	††	38.44 (31.1, 46.2)	9.62 (0.2, 19.0)	1.08 (0.9, 1.3)	††
30-44	4.44 (3.5, 5.6)	-1.06 (-2.3, 0.2)	0.59 (0.4, 0.8)	††	29.94 (24.1, 36.3)	-4.37 (-11.8, 3.1)	0.82 (0.6, 1.1)	††
45-64	2.08(1.7, 2.5)	-0.16 (-0.8, 0.5)	1.01 (0.7, 1.6)	††	23.75 (19.7, 28.2)	-0.78 (-7.9, 6.3)	1.31 (1.0, 1.8)	†
65+	0.76 (0.4, 1.3)	0.50 (-0.3, 1.3)	1.77 (0.8, 4.1)	†	12.15 (6.4, 20.3)	7.41 (-5.0, 19.8)	1.65 (0.8, 3.5)	†
**Sex**								
Male	4.10 (3.4, 4.9)	0.20 (-1.0, 1.4)	0.81 (0.6, 1.1)	††	31.83 (26.4, 37.7)	3.69 (-4.5, 11.9)	1.03 (0.8, 1.3)	††
Female	4.81 (4.0, 5.7)	-0.66 (-1.8, 0.5)	0.70 (0.5, 0.9)	††	31.01 (26.3, 36.1)	-0.29 (-7.1, 6.5)	0.92 (0.7, 1.2)	††
**Race/Ethnicity**								
Hispanic/Latino	4.42 (3.5, 5.5)	0.37 (-1.1, 1.8)	0.90 (0.6, 1.3)	††	35.03 (28.3, 42.3)	5.37 (-4.1, 14.8)	1.14 (0.9, 1.5)	††
White, NH	4.99 (4.2, 5.9)	-0.55 (-1.7, 0.7)	0.77 (0.6, 1.0)	††	28.45 (24.4, 32.8)	-0.48 (-6.7, 5.7)	0.95 (0.7, 1.2)	††
Black, NH	2.97 (1.4, 5.4)	-0.35 (-2.9, 2.2)	1.00 (0.4, 2.3)	†	25.23 (12.8, 41.5)	10.55 (-15.8, 36.9)	1.31 (0.6, 2.6)	†
Asian, NH	3.8 (2.7, 5.2)	-1.31 (-2.9, 0.3)	0.37 (0.2, 0.7)	††	45.64 (25.4, 56.2)	-16.09 (-30.5, -1.7)	0.52 (0.3, 0.8)	††
AI/AN, NH/PI, 2+, NH	3.26 (1.2, 7.1)	1.10 (-2.6, 4.7)	0.62 (0.2, 1.7)	††	14.41 (5.3, 29.3)	12.51 (-4.5, 29.6)	1.47 (0.8, 2.8)	†
**Annual household income**								
<20,000	5.92 (3.8, 8.8)	-0.83 (-3.8, 2.1)	0.63 (0.4, 1.1)	††	48.62 (33.1, 64.3)	-5.69 (-25.5, 14.1)	0.82 (0.5, 1.3)	††
20,000-39,999	5.91 (4.3, 7.9)	1.21 (-1.3, 3.7)	1.01 (0.6, 1.6)	††	38.41 (28.6, 48.9)	4.85 (-8.5, 18.2)	1.48 (1.1, 1.9)	†
40,000-74,999	4.97 (3.5, 6.8)	-0.05 (-2.1, 2.0)	0.77 (0.5, 1.2)	††	31.28 (23.1, 40.4)	7.61 (-3.9, 19.1)	1.11 (0.8, 1.6)	††
75,000-99,999	4.47 (2.9, 6.6)	-0.60 (-2.9, 1.7)	0.70 (0.4, 1.3)	††	31.43 (22.1, 42.0)	-2.56, (-16.1, 11.0)	1.27 (0.8, 1.9)	†
100,000-129,999	5.33 (3.9, 7.1)	-2.45 (-4.3, -0.6)	0.46 (0.3, 0.8)	††	33.57 (23.8, 44.5)	-9.95 (-22.5, 2.5)	0.68 (0.4, 1.1)	††
130,000+	2.21 (1.5, 3.1)	0.63 (-0.5, 1.8)	0.80 (0.5, 1.3)	††	18.86 (13.8, 24.8)	6.15 (-2.5, 14.8)	2.14 (0.9, 5.2)	†††
**Disability**								
Blind/Deaf or Has Severe Vision/Hearing Problem								
Yes	6.5 (3.4, 11.0)	-4.23 (-8.1, -0.3)	0.22 (0.1, 0.6)	††	47.53 (29.1, 66.5)	-27.91 (-51.3, -4.5)	0.58 (0.3, 1.3)	†
No	4.36 (3.8, 5.0)	-0.07 (-0.9, 0.8)	0.79 (0.6, 1.0)	††	30.90 (27.3, 34.7)	2.65 (-2.6, 7.9)	1.02 (0.9, 1.2)	††
Difficulty Concentrating (2019 and later)								
Yes	20.53 (16.5, 25.1)	0.79 (-5.0, 6.6)	. .	. .	50.87 (41.9, 59.8)	-1.53 (-12.8, 9.7)	. .	. .
No	3.37 (2.8, 4.0)	-0.52 (-1.3, 0.3)	. .	. .	27.26 (23.3, 31.5)	0.40 (-5.4, 6.2)	. .	. .
Difficulty Dressing or Bathing (2019 and later)								
Yes	21.4 (8.3, 40.8)	8.78 (-16.3, 33.9)	. .	. .	57.81 (33.1, 80.0)	2.08 (-32.8, 36.9)	. .	. .
No	4.29 (3.7, 4.9)	-0.23 (-1.0, 0.6)	. .	. .	30.83 (27.1, 34.7)	1.62 (-3.6, 6.8)	. .	. .
Difficulty Doing Errands Alone (2019 and later)								
Yes	17.54 (12.4, 23.8)	5.36 (-3.4, 14.1)	. .	. .	50.26 (38.5, 62.0)	1.55 (-15.2, 18.3)	. .	. .
No	4.06 (3.5, 4.7)	-0.32 (-1.2, 0.5)	. .	. .	30.05 (26.2, 34.1)	1.26 (-4.3, 6.8)	. .	. .
Any of the above disabilities	15.06 (12.3, 18.2)	-0.24 (-4.2, 3.7)	. .	. .	50.48 (42.1, 58.8)	-5.20 (-16.0, 5.7)	. .	. .
**Main Occupation (2010 Census Codes)**								
Management, Business, and Financial	4.32 (2.9, 6.2)	-1.66 (-3.5, 0.2)	0.57 (0.3, 1.0)	††	27.68 (19.6, 37.0)	-2.95 (-14.7, 8.8)	0.88 (0.5, 1.5)	††
Computer, Engineering, and Science	4.87 (2.0, 7.3)	-2.17 (-4.5, 0.1)	0.42 (0.2, 0.8)	††	26.16 (16.8, 37.4)	-6.34 (-18.6, 6.0)	0.88 (0.5, 1.5)	†
Education, Legal, Community Service, Arts, and Media	4.31 (2.9, 6.1)	1.32 (-1.0, 3.6)	1.26 (0.7, 2.2)	††	21.87 (15.2, 29.8)	7.95 (-2.5, 18.4)	1.42 (1.0, 2.0)	†
Healthcare Practitioners and Technical	5.01 (2.4, 9.1)	-2.85 (-6.3, 0.6)	0.26 (0.1, 0.8)	††	36.97 (20.5, 56.0)	-21.91 (-42.2, -1.6)	0.28 (0.1, 0.8)	††
Service	5.011 (3.5, 6.9)	0.07 (-2.1, 2.2)	1.01 (0.6, 1.6)	††	41.15 (29.9, 53.1)	-0.80 (-14.5, 12.9)	1.47 (1.1, 1.9)	†
Sales and Related	5.27 (3.4, 7.7)	1.42 (-1.9, 4.7)	0.84 (0.4, 1.6)	††	52.84 (37.7, 67.6)	-9.32 (-28.7, 10.1)	0.72 (0.4, 1.2)	††
Office and Administrative Support	4.71 (3.3, 6.5)	-0.70 (-2.8, 1.4)	1.21 (0.5, 2.8)	†††	31.69 (22.5, 42.1)	-0.31 (-13.5, 12.9)	1.57 (0.7, 3.4)	†††
Farming, Fishing, and Forestry*	. .	. .	. .		. .	. .	. .	†
Construction and Extraction	4.54 (1.6, 9.8)	-2.01 (-6.3, 2.2)	1.16 (0.5, 3.0)	†	38.48 (10.3, 74.0)	-12.63 (-52.2, 27.0)	0.87 (0.3, 2.2)	†
Installation, Maintenance, and Repair	1.21 (0.4, 2.7)	1.73 (-1.4, 4.9)	1.51 (0.5, 4.6)	†	12.90 (1.1, 43.1)	12.96 (-18.0, 43.9)	1.26 (0.4, 3.5)	†
Production	2.72 (0.4, 8.7)	1.57 (-2.4, 5.6)	0.81 (0.3, 2.3)	††	37.07 (4.2, 83.9)	4.42 (-44.0, 52.8)	1.86 (1.0, 3.6)	†
Transportation and Material Moving	4.49 (1.5, 10.1)	0.50 (-4.6, 5.6)	0.64 (0.2, 1.8)	††	28.80 (10.3, 54.6)	24.51 (-6.2, 55.3)	1.79 (1.0, 3.1)	†

Notes: † designates intercept, †† designates linear, ††† designates quadratic; NH refers to Non-Hispanic; **Farming, Fishing, and Forestry Occupations had their suicidal ideation results suppressed due to small cell sizes*

Supplemental Figure S1. Depressed mood in the past 30 days from 2013 to 2020 by age category.

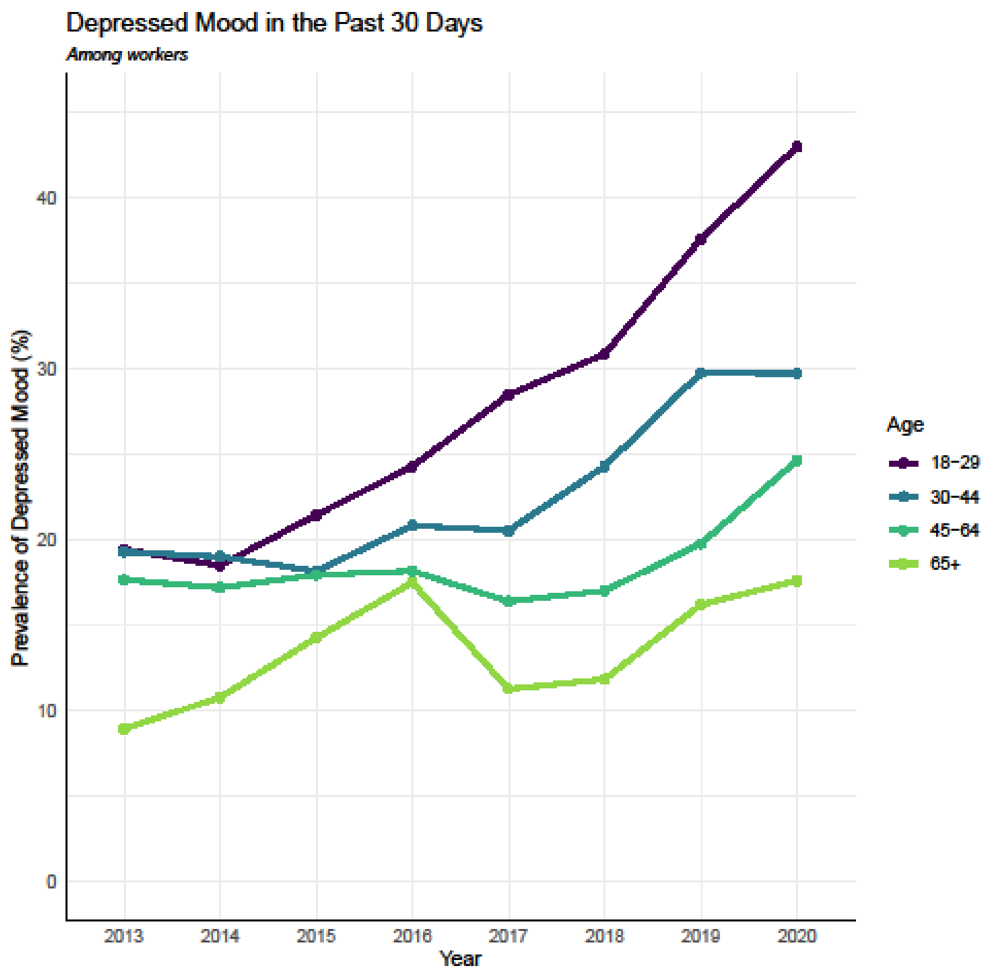

Supplement: Supplementary file 1 [file ijerph-20-01253-s001.zip › ijerph-2105033-supplementary.pdf]
